# Modeling of Electron‐Transfer Kinetics in Magnesium Electrolytes: Influence of the Solvent on the Battery Performance

**DOI:** 10.1002/cssc.202101498

**Published:** 2021-10-07

**Authors:** Janina Drews, Piotr Jankowski, Joachim Häcker, Zhenyou Li, Timo Danner, Juan Maria García Lastra, Tejs Vegge, Norbert Wagner, K. Andreas Friedrich, Zhirong Zhao‐Karger, Maximilian Fichtner, Arnulf Latz

**Affiliations:** ^1^ Institute of Engineering Thermodynamics, German Aerospace Center (DLR) Pfaffenwaldring 38–40 70569 Stuttgart Germany; ^2^ Helmholtz Institute Ulm (HIU) Helmholtzstr.11 89081 Ulm Germany; ^3^ Department of Energy Conversion and Storage Technical University of Denmark (DTU) Anker Engelunds Vej 2800 Kgs. Lyngby Denmark; ^4^ Faculty of Chemistry Warsaw University of Technology (WUT) Noakowskiego 3 00661 Warsaw Poland; ^5^ Institute of Nanotechnology Karlsruhe Institute of Technology (KIT) Hermann-von-Helmholtz-Platz 1 76344 Eggenstein-Leopoldshafen Germany; ^6^ Institute of Energy Storage University of Stuttgart Pfaffenwaldring 31 70569 Stuttgart Germany; ^7^ Institute of Electrochemistry Ulm University (UUlm) Albert-Einstein-Allee 47 89081 Ulm Germany

**Keywords:** Computational chemistry, Deposition mechanism, Desolvation, Kinetics, Rechargeable magnesium batteries

## Abstract

The performance of rechargeable magnesium batteries is strongly dependent on the choice of electrolyte. The desolvation of multivalent cations usually goes along with high energy barriers, which can have a crucial impact on the plating reaction. This can lead to significantly higher overpotentials for magnesium deposition compared to magnesium dissolution. In this work we combine experimental measurements with DFT calculations and continuum modelling to analyze Mg deposition in various solvents. Jointly, these methods provide a better understanding of the electrode reactions and especially the magnesium deposition mechanism. Thereby, a kinetic model for electrochemical reactions at metal electrodes is developed, which explicitly couples desolvation to electron transfer and, furthermore, qualitatively takes into account effects of the electrochemical double layer. The influence of different solvents on the battery performance is studied for the state‐of‐the‐art magnesium tetrakis(hexafluoroisopropyloxy)borate electrolyte salt. It becomes apparent that not necessarily a whole solvent molecule must be stripped from the solvated magnesium cation before the first reduction step can take place. For Mg reduction it seems to be sufficient to have one coordination site available, so that the magnesium cation is able to get closer to the electrode surface. Thereby, the initial desolvation of the magnesium cation determines the deposition reaction for mono‐, tri‐ and tetraglyme, whereas the influence of the desolvation on the plating reaction is minor for diglyme and tetrahydrofuran. Overall, we can give a clear recommendation for diglyme to be applied as solvent in magnesium electrolytes.

## Graphical abstract

**Desolvation vs. electron transfer**: The combination of DFT and continuum modeling techniques with experimental measurements sheds light on the magnesium deposition mechanism and the role of the involved solvent. Thereby, a new kinetic model for the electrochemical reaction is developed and general properties, which are crucial for fast kinetics and low overpotentials, are identified.

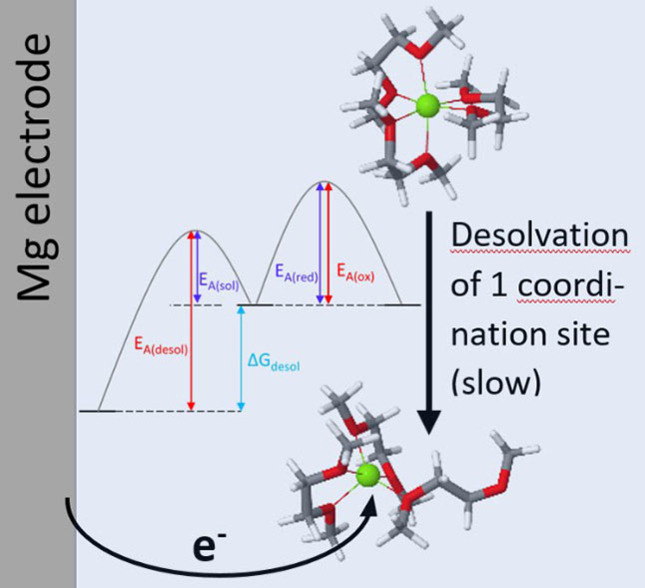

## Introduction

Regarding energy density, safety, cost and sustainability rechargeable magnesium batteries are a very promising next‐generation energy storage technology. On the one hand the natural abundance of magnesium enables sustainable and economic large‐scale applications.[^1^, ^2^, ^3^, ^4^] On the other hand magnesium is less prone to dendrite formation than many other metals.[^5^, ^6^] The possibility to use metal anodes combined with the bivalency of the cationic charge carriers are key to high specific capacities.[^1^, ^2^, ^7^, ^8^]

However, the bivalent nature of the cations is a prominent advantage and a big challenge of magnesium batteries at the same time. The high charge density is the basis for a very high volumetric capacity but it also leads to strong coulomb interactions with anions as well as solvent molecules. This causes high kinetic barriers for desolvation and solid‐state diffusion. Latter hinders the intercalation reaction and reduces the mobility of magnesium cations in surface films, which leads to a strong tendency of the magnesium anode for passivation.[^1^, ^8^, ^9^] The other issue resulting from the bivalency of magnesium is that due to the strong electrostatic attraction of anions a good solvation is important for the dissociation of magnesium salts. This is in turn crucial for a high ionic conductivity of the electrolyte.[^2^, ^10^, ^11^, ^12^, ^13^, ^14^] At the same time the desolvation of magnesium ions close to the electrode surface plays an important role during the deposition or intercalation process.[^1^, ^9^, ^15^, ^16^, ^17^, ^18^] Since a sluggish desolvation can kinetically hinder the charge transfer reaction, the solvation of the magnesium cation should only be as good as necessary. Consequently, the choice of both – solvent and anion – is crucial for the performance of magnesium batteries.

For instance, it is known that the presence of chloride anions in the electrolyte facilitates the desolvation and therefore the magnesium deposition.[^1^, ^9^, ^19^, ^20^] As a result chloride containing electrolytes are commonly used for magnesium batteries. But since chlorides also cause corrosion, lots of research has been done recently to develop chloride‐free magnesium electrolytes, which can provide a similar or even better performance than chloride containing ones.[^1^, ^21^, ^22^, ^23^, ^24^, ^25^] One very promising electrolyte with high ionic conductivity and a wide electrochemical stability window is based on the non‐nucleophilic magnesium tetrakis(hexafluoroisopropyloxy)borate salt Mg[B(hfip)_4_]_2_.^[26]^ Thereby, the typical solvent for this state‐of‐the‐art chloride‐free electrolyte is dimethoxyethane (DME/G1).[^25^, ^26^, ^27^, ^28^, ^29^] In general, ethereal solvents are commonly used for magnesium batteries since they provide the required stability against the magnesium metal anode.[^1^, ^30^] Apart from DME, tetrahydrofuran (THF) is a very popular solvent especially for chloride‐containing electrolytes.[^8^, ^9^] However, both DME and THF are rather volatile and highly flammable, which is an issue regarding the safety of magnesium batteries.^[2]^ Therefore, longer glymes CH_3_O−(C_2_H_4_O)n−CH_3_ (Gn) with n>1 are considered more and more frequently as solvents for magnesium batteries.[^20^, ^25^, ^31^, ^32^, ^33^] The multidenticity of glymes is beneficial for the solubility of magnesium salts, but especially the higher order glymes also significantly reduce the mobility of the ions due to their higher viscosity.^[11]^ Moreover, the properties of the solvent and the structure of the electrochemically active specie can significantly impact the desolvation behaviour and consequently the magnesium deposition.

Mathematical descriptions of the kinetics at the electrode‐electrolyte interface provide a relation between the Faradaic current and overpotential across the interface. Thereby, the electrochemical kinetics are commonly described by the simple Butler‐Volmer equation.[^34^, ^35^, ^36^, ^37^] The symmetry factor allows to take into account, that the stripping and plating reaction can have different contributions to the overpotential. Nevertheless for many systems it is observed that the anodic and cathodic reactions are (almost) symmetric.[^35^, ^38^] However, for multivalent ions this must not be the case, since the electron transfer does not have to occur as a single step reaction and other processes like desolvation might limit the metal deposition. In the case of magnesium and DME based chloride‐free electrolytes the cathodic symmetry factor seems to be significantly smaller than 0.5.^[39]^ Consequently, the transition state during plating from DME solvated magnesium cations is more reactant‐ than product‐like, which indicates that the desolvation plays an important role during the magnesium deposition. Therefore, the solvent (and anion) might significantly influence the symmetry factor of the Butler‐Volmer kinetics. Since this parameter is of phenomenological nature,^[40]^ it is not straightforward to predict, how it changes with the electrolyte composition. Moreover, the Butler‐Volmer equation is not easily applicable when the electrolyte contains more than one electrochemically active species.

Density functional theory (DFT) is a powerful method to investigate the stability and reactivity of electrolyte species, which can provide important insights into reaction mechanisms and the corresponding intermediate species, e. g. regarding clustering, desolvation, intercalation, electron transfer and decomposition.[^6^, ^11^, ^20^, ^41^, ^42^] Therefore, DFT modeling contributes to a better understanding of the processes during battery operation on a molecular scale, which in turn should be included into continuum work to get a more holistic picture.

This work combines DFT calculations and continuum modelling with experimental measurements with the aim to provide comprehensive insights about the influence of different solvents on the battery performance, especially regarding the role of desolvation during magnesium deposition. In a first step, DFT calculations are used to determine the mechanism of magnesium deposition, which enables identification of the rate determining step, the corresponding energy barrier as well as the relevant intermediate specie. On this basis, a model for the electron‐transfer kinetics is developed and parametrized, which explicitly considers the characteristics of multivalent cations like magnesium. As seen from the DFT results, the initial desolvation of the cation, which is required, before it can be reduced, plays a crucial role and therefore, has to be included into the description of the electrochemical kinetics. Moreover, the qualitative effect of the electrochemicial double layer is considered during parametrization of the model. The kinetic model is then used to screen different solvents (e. g. THF and glymes) for a Mg[B(hfip)_4_]_2_ electrolyte with the aim to explain and predict, which solvent is most beneficial for the battery performance.

## Methodology

### DFT Calculations

DFT calulcations were performed using the M06‐2X functional and 6‐311++G(d,p) basis set as implemented in Gaussian16 package.^[43]^ The effect of a liquid surrounding was modelled using conductor‐like polarizable continuum model (CPCM) for electron transfer reactions, and to account for solvation entropy when calculating desolvation energies, we switched to the Solvation Model based on Density (SMD). In both cases, parameters for THF (ϵ=7.4257 ) were used, following the previous study.^[20]^ All geometries were optimized and their local minimum configuration was confirmed by the absence of imaginary modes in the frequency calculations. The desolvation energies were obtained by step‐by‐step removal of single oxygen atoms from the cation solvation shell, and calculated as a change in Gibbs free energy. The desolvation procedure was initiated from the fully solvated structures of Mg(G1)_3_^2+^ and Mg(G2)_2_^2+^, with coordination numbers of six, as found before to be the most stable,^[20]^ and then the number of oxygen atoms in the first coordination shell was gradually decreased by one each time. At least four different starting geometries were created at each of the steps, and re‐optimized. Only the structures with the lowest energy were further considered in the study. To assess the ability for electron transfer, an extra electron was added to such obtained structures, and the structures were relaxed. The reduction potentials were calculated based on thermodynamic cycle of the electrode reaction,^[44]^ and converted from absolute potentials to the Mg^2+^/Mg scale by 2.14 V.^[45]^ The ionic radii of the complexes was calculated as the radii of the smallest sphere that can fit the DFT optimized geometry, considering each of the atoms as van der Waals spheres.

### Experiments

Electrolyte solutions with a concentration of 0.2 M and 0.3 M Mg[B(hfip)_4_]_2_ in different organic solvents were prepared under argon atmosphere (O_2_<1 ppm, H_2_O<1 ppm). Therefore, Mg[B(hfip)_4_]_2_ powder, synthesized from a two‐step reaction according to our previous work,^[26]^ is dissoluted in ethylene glycol dimethyl ether (G1, monoglyme, 99.5 %, <10 ppm H_2_O, AcrosOrganics), diethylene glycol dimethyl ether (G2, diglyme, 99+ %, <10 ppm H_2_O, AcrosOrganics), tetraethylene glycol dimethyl ether (G4, tetraglyme, 99 %, <10 ppm H_2_O, AcrosOrganics) and tetrahydrofuran (THF, <10 ppm H_2_O, Sigma‐Aldrich). All solvents were stored over molecular sieve. After stirring for 24 h, the solutions were filtered and used as electrolytes for cell assembly in an argon‐filled glove box.

Cycling experiments in a two‐electrode Mg−Mg symmetric cell setup with two borosilicate glass fiber GF/C separators and 0.3 M electrolyte solution in G1 and G2 were conducted to investigate the long‐term overpotential evolution. The cell was tested with a current density of 1 mA cm^−2^ until an area capacity of 1 mAh cm^−2^ was reached. Complementary, cyclic voltammetry was performed with a Swagelok cell at a scan rate of 50 mV s^−1^ to analyze the reversible redox peak positions. Therefore, a two‐electrode cell with Pt as working and Mg as counter electrode was utilized. The measurements were conducted using a Biologic VMP‐3 potentiostat. The operating temperature was 25±0.1 °C.

In addition, polarization experiments were conducted applying ECC‐PAT‐Core cells from EL‐CELL^TM^ containing a ring‐shaped magnesium metal foil as reference electrode (RE). The cell comprises two layers of glass fiber separator (260 μm, GF/C, Whatman) and 200 μL 0.2 M electrolyte solution. Magnesium metal foil (100 μm, 99.5 %, Gelon) was used as working (WE) and counter (CE) electrode, with both electrodes being scraped beforehand under argon atmosphere to remove the native surface oxide layer. After cell assembly, the cells were held for 50 h at OCV before being alternately polarized for ten cycles vs. Mg‐RE with a current rate of 0.1, 0.2, 0.5 and 1.0 mA cm^−2^. In each stripping/plating step a charge depth of 0.2 mAh cm^−2^ was realized with 10 min and 10 h rest time in between and after 10 cycles, respectively. Besides the half‐cell potential (WE‐RE), also the full cell voltage (WE‐CE) is logged. Thus, a differentiation between stripping and plating is feasible and overpotential asymmetries can be identified.

### Continuum model

#### Kinetic model

**Figure 1:**
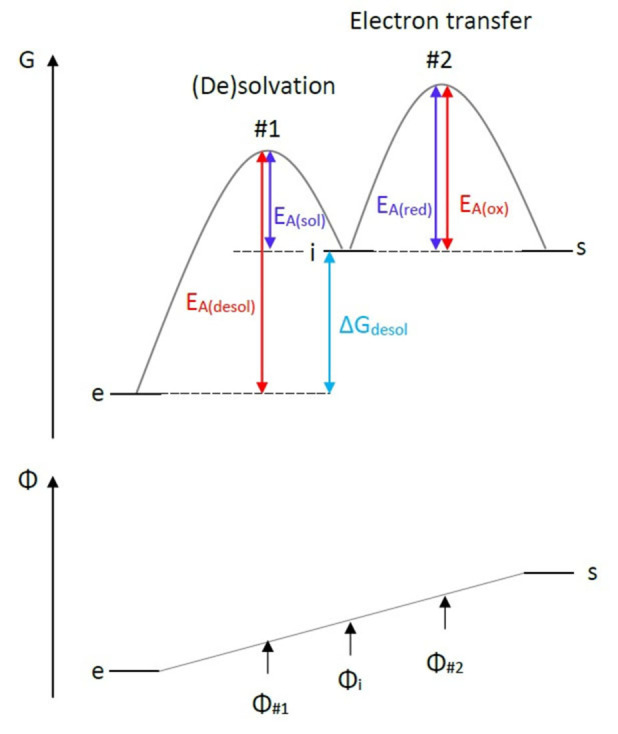
Energy diagram for (de)solvation and electron transfer at the electrode‐electrolyte interface as well as corresponding electric potentials for the solvated magnesium cation in the electrolyte (e), the (partially) desolvated intermediate (i), the solid magnesium of the electrode (s) and the two transition states (#1 and #2).

For magnesium the desolvation is considered to be very important during the deposition process. Consequently, the electrochemical reaction at the electrode‐electrolyte interface can not be regarded as a simple one‐step reaction, but has to be described by at least two subsequent steps: The (de)solvation and the electron transfer reaction (Figure 1). Note, that both processes itself also include more than one step: The (de)solvation has to occur at multiple coordination sites of the magnesium cation and furthermore two electrons have to be transferred. The details of this complex multi‐step deposition are analyzed by DFT calculations (cf. Section Magnesium deposition mechanism and Figure 5). As indicated by Figure 1 the kinetic model simplifies both the (de)solvation and the electron transfer to single step reactions, whereby the reactions rates are considered as effective quantities determined by the corresponding rate‐determining sub‐step.

The total reaction rate and consequently the current, which flows across the electrode‐electrolyte interface, will be determined by the slowest step of the forward and backward reaction respectively. Thereby, the DFT calculations indicate that the desolvation is the rate‐limiting step for magnesium deposition (cf. Section Magnesium deposition mechanism and Figure 5), whereas the electron transfer determines the rate of magnesium dissolution (Figure 1). Consequently, the current density $i_{\mathrm{se}}$ at the interface (se) between the solid electrode (s) and the electrolyte (e) can be described by the following approximation:

(1) $$i_{\mathrm{se}}=z_{+}F\cdot \left(\nu_{\mathrm{ox}}-\sum_{j}\nu_{\mathrm{desol}\;\left(j\right)}\right)$$

whereby $z_{+}=2$ is the number of transfered electrons, *F* is the Faraday constant, *j* describes the number of electroactive species and $v_{\mathrm{ox}}$ and $v_{\mathrm{desol}}$ are the reaction rates of the oxidation and the desolvation reaction, respectively. Since the desolvation barrier can differ significantly in different electrolytes it has to be mentioned that our simplification is only valid for large desolvation energy barriers. Once the reaction rates for desolvation and electron transfer are in the same order of magnitude a more detailed kinetic mechanism taking into account both processes has to be considered. This is discussed in more detail in Section S1.2.3 of the Supporting Information.

Due to ion pairing or clustering, the electrolyte might contain more than one electroactive specie (*j*>1) especially in the case of chloride containing electrolytes.[^10^, ^11^, ^12^, ^13^, ^19^, ^31^, ^32^, ^46^, ^47^] However, the B(hfip)4^−^ anion is very bulky and weakly coordinating, which facilitates the dissociation of the chloride‐free magnesium salt.^[26]^ Moreover, the redissociation phenomenon favors the formation of free charge carriers at non‐dilute concentrations.^[32]^ Therefore, ion pairing can be neglected for medium concentrated Mg[B(hfip)_4_]_2_ electrolytes. Also the formation of bigger clusters due to entropic effects, which can happen at high concentrations close to the solubility limit, is neglectable for electrolyte concentrations smaller than 0.35 M.^[48]^ The simulations will focus on 0.2 M electrolytes, in which the Mg[B(hfip)_4_]_2_ salt is highly dissociated and the fully solvated magnesium cations are the main electrochemically active specie.[^18^, ^26^, ^49^, ^50^] Consequently, only one specie has to be considered in the kinetic model (*j*=1) and Equation (1) simplifies to Equation 2:

(2) $$i_{\mathrm{se}}=z_{+}F\cdot \left(v_{\mathrm{ox}}-v_{\mathrm{desol}}\right)$$

Since the electron transfer is regarded as rate‐determining step for the stripping reaction (Figure 5), the anion and solvent can not have a big impact on this part of the electrochemical reaction. Therefore, the magnesium dissolution is considered to be independent of the number of electroactive species [Eqs. (1) and 2].

(3) $${\underbrace{\mathrm{Mg}}}_{s}\to {\mathrm{Mg}}^{2+}+2e^{-}$$

The opposite is true for the plating reaction, which is found to be limited by the desolvation reaction (Figure 5). For magnesium deposition the fully solvated electroactive specie (e) has to be (partially) desolvated into an intermediate (i), which consequently has one or more free coordination sites. Therefore, the (partially) desolvated magnesium cation is able to get closer to the electrode surface than the fully solvated one. Moreover, a physical or chemical bond might be formed between the intermediate cation and the negatively charged metal surface. This spatial proximity of the magnesium cation and the electrode enables the electron transfer reaction so that solid magnesium (s) can be deposited. In fully dissociated, chlorine‐free electrolytes like medium concentrated Mg[B(hfip)_4_]_2_ solutions the desolvation, which is required for the reduction of the magnesium cation, can be described by Equation 4:

(4) $${\underbrace{\mathrm{Mg}{\left(\mathrm{Sol}\right)}_{X}^{2+}}}_{e}\to {\underbrace{\mathrm{Mg}{\left(\mathrm{Sol}\right)}_{X-W}^{2+}}}_{i}+w\;\mathrm{Sol}$$

where Sol denotes the solvent, *x* is the number of solvent molecules in the solvation shell of the fully solvated electroactive specie, and *w* describes, how many solvent molecules need to be desolvated prior to the electron transfer. The rate equation of a reaction is given by the rate constant *k* and the concentrations of the reactants *c*, whereby the stoichiometry has to be considered.^[51]^ Moreover, for sterical reasons only a limited number of the (partially) desolvated intermediates ($c_{i}<c_{i,\max}$ ) is able to get close to the electrode surface, which is required for the subsequent electron transfer reaction. The result is an adsorption‐like kinetic law [Eq. 5]:

(5) $$v_{\mathrm{desol}}=k_{\mathrm{desol}}\cdot c_{i,\max}\cdot \left(1-\frac{c_{i}}{c_{i,\max}}\right)\cdot c_{e}$$

Thereby, the Arrhenius equation relates the rate constant to the activation energy *E*_A_ of the reaction:

(6) $$k=A\cdot \exp\left(-\frac{E_{A}}{RT}\right)$$

Since the desolvation has to happen close to the electrode surface to enable magnesium deposition, the fully solvated magnesium and the partially solvated intermediate experience the gradient of the electric potential Φ in the electrochemical double layer (Figure 1). Therefore, the activation energy can be divided into a chemical and an electric part. It can be written as the difference of the electrochemical potentials $\tilde{\mu }=\mu +zF\Phi$ of the corresponding transition state #1 and the fully solvated electroactive specie (e):

(7) $$E_{A\;\left(\mathrm{desol}\right)}={\tilde{\mu }}_{\#1}-{\tilde{\mu }}_{e}\;\;\;\;={\underbrace{\mu_{\#1}-\mu_{e}}}_{E_{A\;\left(\mathrm{desolv}\right)}^{\mathrm{chemical}}}+{\underbrace{z_{\#1}F\Phi_{\#1}-z_{e}F\Phi_{e}}}_{E_{A\;\left(\mathrm{desolv}\right)}^{\mathrm{electric}}}$$

The electric potential, which is experienced by the intermediate ($\Phi_{i}$ ), can be defined relative to the potential in the electrolyte $\Phi_{e}$ and the potential difference between electrode and electrolyte, whereby $0\leq \alpha_{i}\leq 1$ describes, to what extent the potential difference in the electrochemical double layer influences the (de)solvation reaction.

(8) $$\Phi_{i}=\Phi_{e}+\alpha_{i}\cdot \left(\Phi_{s}-\Phi_{e}\right)$$

An analogue expression can be used to describe the electric potential of the transition state $\Phi_{\#1}$ :

(9) $$\Phi_{\#1}=\Phi_{e}+\alpha_{\#1}\cdot \left(\Phi_{i}-\Phi_{e}\right)$$

where $\alpha_{\#1}$ is the symmetry factor of the desolvation reaction, which indicates whether the transition state is more reactant‐ or product‐like. Since no charge transfer happens during the (de)solvation, the charge of the fully and partially solvated magnesium as well as the charge of the transition state are equal ($z_{e}=z_{\#1}=z_{i}=2$ ). Moreover, the chemical rate constant can be defined by Equation 10:

(10) $$k^{0}=A\cdot \exp\left(-\frac{E_{A}^{\mathrm{chemical}}}{RT}\right)$$

Combing Equation (5)–(10) finally results in following rate equation for the desolvation:

(11) $$v_{\mathrm{desol}}=k_{\mathrm{desol}}^{0}\cdot c_{i,\max}\cdot \left(1-\frac{c_{i}}{c_{i,\max}}\right)\cdot c_{e}\cdot \exp\left(-\frac{z_{e}F}{RT}\alpha_{\#1}\alpha_{i}\left(\Phi_{s}-\Phi_{e}\right)\right)$$

Since the desolvation is assumed to be the slowest step during magnesium deposition, the concentration of the (partially) desolvated intermediate will always be extremely small ($c_{i}\ll c_{i,max})$ . Consequently Equation (11) simplifies to Equation 12:

(12) $$v_{\mathrm{desol}}={\underbrace{k_{\mathrm{desol}}^{0}\cdot c_{i,\max}}}_{K_{\mathrm{desol}}^{0}}\cdot c_{e}\cdot \exp\left(-\frac{z_{e}F}{RT}\alpha_{\#1}\alpha_{i}\left(\Phi_{s}-\Phi_{e}\right)\right)$$

The kinetics of the oxidation reaction can be described by the standard Butler‐Volmer approach.[^34^, ^52^] For $z_{+}=2$ transfered electrons the activation energy is given by Equation 13:

(13) $$E_{A(\mathrm{ox})}=E_{A(\mathrm{ox})}^{\mathrm{chemical}}-z_{+}F\cdot \left(1-\alpha_{\#2}\right)\left(1-\alpha_{i}\right)\left(\Phi_{s}-\Phi_{e}\right)$$

where $\alpha_{\#2}$ is the cathodic symmetry factor, which describes the electric potential experienced by the transition state #2: $\Phi_{\#2}=\Phi_{i}+\alpha_{\#2}\cdot \left(\Phi_{s}-\Phi_{i}\right)$ . Equation (6) and (10) relate the activation energy [Eq. (13)] to the reaction rate of the electron transfer reaction [Eq. 14].

(14) $$v_{\mathrm{ox}}={\underbrace{k_{\mathrm{ox}}^{0}\cdot c_{s}}}_{K_{\mathrm{ox}}^{0}}\cdot \exp\left(\frac{z_{+}F}{RT}\left(1-\alpha_{\#2}\right)\left(1-\alpha_{i}\right)\left(\Phi_{s}-\Phi_{e}\right)\right)$$

Equation (2), (12) and (14) finally lead to following expression for the current density across the interface:

(15) $$i_{\mathrm{se}}=z_{+}F\cdot \left[K_{\mathrm{ox}}^{0}\cdot \exp\left(\frac{z_{+}F}{RT}\left(1-\alpha_{\#2}\right)\left(1-\alpha_{i}\right)\left(\Phi_{s}-\Phi_{e}\right)\right)\right.\left.-K_{\mathrm{desolv}}^{0}\cdot c_{e}\cdot \exp\left(-\frac{z_{e}F}{RT}\alpha_{\#1}\alpha_{i}\left(\Phi_{s}-\Phi_{e}\right)\right)\right]$$

Finally, one should bear in mind, that the above describe kinetic model [Eq. (15)] is only valid, when the desolvation of the electroactive cation is significantly slower than its reduction. Moreover, a detailed analysis of the equilibrium state and the influence of the solvent on the half‐cell potential can be found in the Supporting Information (cf. Section S1.2.3).

#### Transport model

The above described kinetic model for the desolvation and electron transfer reaction [Eq. (15)] is coupled to our general transport theory [Eqs. (S8)–(S10) in the Supporting Information], which was presented in earlier work.^[36]^ The equation system is simplified for an isothermal process (T=298.15 K) and solved for the magnesium salt concentration $c_{\pm }$ as well as for the electric potentials of the electrolyte $\Phi_{e}$ and the two electrodes $\Phi_{s}$ of a symmetric magnesium cell. To analyze the half cell potentials during plating and stripping, a reference potential in the middle of the electrochemical cell has to be determined. Since the reference measurement is currentless $i_{\mathrm{se}}^{\mathrm{ref}}=0$ , the potential $\Phi_{s}^{\mathrm{ref}}$ of a magnesium reference electrode can be determined by using the same interface model as for the working electrodes [Eq. (15)].

#### Model parameters

In general model parameters are derived from our DFT simulations and experiments. In this study G1 serves as reference material and we determine kinetic parameters in our electrochemical measurements using G1 as solvent. Missing properties are calculated relative to the G1 system which allows us to deduce qualitative trends and predictions. Note, that the solvent affects both the transport in the electrolyte as well as the reaction at the electrodes.

Since there is no concentration‐dependent experimental data of the transport parameters for all analyzed solvents, they are taken to be constant (Table 1). Moreover, it is assumed, that the transference number does not differ significantly in the analyzed solvents. The diffusion coefficients are referred to the one in G1 ($D_{G1}\approx 10^{-10}$ m^2^ s^−1^). Via the Stokes‐Einstein equation an inverse dependence on the viscosity *η* as well as the hydrodynamic radius of the fully solvated magnesium cation *r_e_* is considered [Eq. 16]:

(16) $$\frac{D_{\mathrm{sol}}}{D_{G1}}=\frac{\eta_{G1}\cdot r_{e\left(G1\right)}}{\eta_{\mathrm{sol}}\cdot r_{e\left(\mathrm{sol}\right)}}$$

**Table 1:** Solvent properties and transport parameters for a 0.2 M Mg[B(hfip)_4_]_2_ electrolyte.

Parameter	G1	G2	G3	G4	THF	Source
Solvent properties						
$\epsilon_{r}$	7.2	7.4	7.6	7.8	7.6	[^53^, ^54^, ^55^]
*ρ* [g cm^−3^]	0.865	0.939	0.981	1.007	0.881	[^53^, ^54^, ^55^]
*c* ^0^ [mol L^−1^]	9.60	7.00	5.50	4.53	12.22	–
*η* [mPa s]	0.557	1.028	1.96	3.275	0.452	[^56^, ^57^] ${}^{\left[b\right]}$
$\eta_{0.2M}$ [mPa s]	0.741	2.039	‐	6.178	1.081	${}^{\left[b\right]}$

Transport parameters						
*x*	3	2	2	2	6	^[20]^ ${}^{\left[c\right]}$
*r*_e_ [Å]	4.391	4.433	5.340	6.730	5.668	${}^{\left[c\right]}$
$\kappa_{0.2M}$ [S m ^−1^]	0.9990	0.3711	0.2682${}^{\left[a\right]}$	0.1652	0.544	${}^{\left[b\right]}$
$D_{\mathrm{sol}}/D_{G1}$	1.000	0.360	0.234	0.078	0.53	
$t_{+}$	0.185	0.185	0.185	0.185	0.185	${}^{\left[b\right]}$

[a] Mean of the data of G2 and G4. [b] Experimental measurements. [c] Results of DFT calculations.

A summary of all solvent and transport parameters is given in Table 1.

The parameters of the kinetic model $K_{\mathrm{ox}}^{0}$ , $K_{\mathrm{des}}^{0}$ , and $\alpha_{i}$ cannot be directly determined by atomistic simulations or dedicated measurements. Additional assumptions are made for the approximation of $\alpha_{i}$ and, finally, the rate constants of the reactions are determined to reproduce the experimental data measured using G1. Details of this procedure are given below and in Section Rate constants of the desolvation and oxidation reactions.

We assume, that the two transition states during the desolvation and the oxidation reaction are symmetric ($\alpha_{\#1}=\alpha_{\#2}=0.5$ ). Moreover, the impact of the solvent on the magnesium dissolution can be neglected. Therefore, the same stripping rate constant $K_{\mathrm{ox}}^{0}$ can be used for all solvents. This topic will be revisited in the paragraph below. In contrast, the rate constant for the desolvation reaction $K_{\mathrm{desol}}^{0}$ will be strongly influenced by the solvent. More precisely, $K_{\mathrm{desol}}^{0}$ depends on the strength of solvent‐cation interactions as well as on the steric demands of the (partially) desolvated intermediate [Eq. (12)]. Both effects are considered relative to G1. The maximum concentration of intermediate, which can be spatially close enough to the electrode surface, is determined by the number of (partially) desolvated magnesium cations, that fit into a monolayer. Consequently, the steric effects can be considered via the radius of the intermediate *r*_i_:

(17) $$\frac{c_{i,\max\;\left(\mathrm{Sol}\right)}}{c_{i,\max\;\left(G1\right)}}=\frac{r_{i\left(G1\right)}^{2}}{r_{i\left(\mathrm{Sol}\right)}^{2}}$$

The chemical rate constants [Eq. (10)] are related to each other by the Arrhenius equation:

(18) $$\frac{k_{\mathrm{desol}\;\left(\mathrm{Sol}\right)}^{0}}{k_{\mathrm{desol}\;\left(G1\right)}^{0}}=\frac{A_{\mathrm{Sol}}}{A_{G1}}\cdot \exp\left(\frac{E_{A,\mathrm{desol}\;\left(G1\right)}^{\mathrm{chemical}}-E_{A,\mathrm{desol}\;\left(\mathrm{Sol}\right)}^{\mathrm{chemical}}}{RT}\right)$$

Thereby, the activation energy for the desolvation $E_{A,\mathrm{desol}}$ is directly related to the required desolvation energy $\Delta G_{\mathrm{desol}}$ (Figure 1):

(19) $$E_{A,\mathrm{desol}}=E_{A,\mathrm{sol}}+\Delta G_{\mathrm{desol}}$$

Moreover, it can be assumed, that the pre‐exponential factor as well as the activation energy for the solvation is quite similar for all solvents ($A_{\mathrm{sol}}\approx A_{G1}$ and *E*_A,sol  (Sol)_≈*E*_A,sol (G1)_). Additionally, the desolvation energy should be significantly higher than the activation energy for the solvation ($\Delta G_{\mathrm{desol}}\gg E_{A,\mathrm{sol}}$ ). Consequently, Equation (18) and (19) result in following relation between the chemical part of the desolvation rate constants:

(20) $$\frac{k_{\mathrm{desol}\;\left(\mathrm{Sol}\right)}^{0}}{k_{\mathrm{desol}\;\left(G1\right)}^{0}}\approx \exp\left(\frac{\Delta G_{\mathrm{desol}\;\left(G1\right)}-\Delta G_{\mathrm{desol}\;\left(\mathrm{Sol}\right)}}{RT}\right)$$

**Figure 2:**
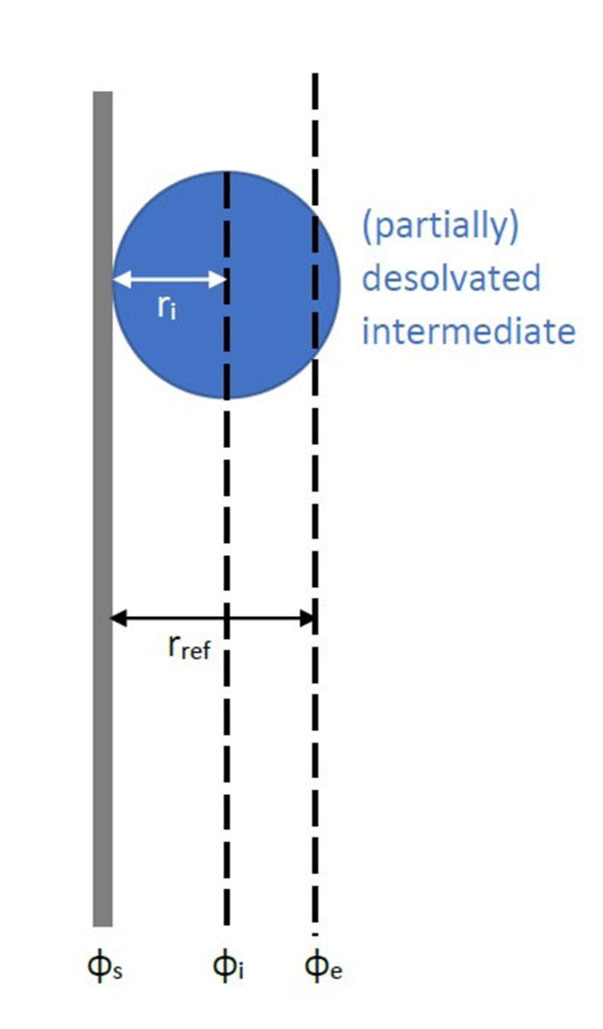
Determination of *α_i_* on the basis of a linear potential decay in the electrochemical double layer.

Since $\alpha_{i}$ is closely related to the potential decay in the electrochemical double layer, it is the second parameter of the kinetic model, which strongly depends on the solvent. Thereby, the size of the fully and partially solvated magnesium cations as well as the dielectric constant of the solvent will impact $\alpha_{i}$ depending on the structure of the electrochemical double layer. As a first approach, it is assumed, that the electric potential decreases linearly in the double layer. Moreover, the (partially) desolvated intermediate is described as a sphere, which experiences the potential at its center. This simple assumption leads to following expression for $\alpha_{i}$ (Figure 2):

(21) $$\alpha_{i}=\frac{\Phi_{i}}{\Phi_{s}-\Phi_{e}}\approx \frac{r_{\mathrm{ref}}-r_{i}}{r_{\mathrm{ref}}}$$

where $r_{\mathrm{ref}}$ describes the distance from the electrode surface, at which the electric potential has dropped to the value of the bulk electrolyte.

The reference length $r_{\mathrm{ref}}$ can be estimated via the Debye length $\lambda_{D}$ . For multivalent ions it is known, that the actual Debye length is longer than expected.^[58]^ One possible reason for this might be that electrolytes, which contain multivalent ions, don't necessarily behave like ideal solutions especially in the electrochemical double layer, where the ionic concentrations can become quite high. Therefore, it is suggested to use activities (a=γ·c ) instead of concentrations for determining the effective Debye length $\lambda_{D,\mathrm{eff}}$ :^[59]^

(22) $$\lambda_{D,\mathrm{eff}}=\sqrt{\frac{\epsilon_{0}\epsilon_{r}k_{B}T}{2N_{A}e^{2}\cdot 0.5\sum_{j}z_{j}^{2}a_{j}}}$$

Thereby, the modified Davies equation is used to describe the activity coefficients of the dissolved ions [Eqs. (S1)–(S4)].^[60]^ Since steric effects also impact the extent of the electrochemical double layer, the reference length $r_{\mathrm{ref}}$ is defined as sum of the effective Debye length and the radius of the fully solvated magnesium cation *r*_e_:

(23) $$r_{\mathrm{ref}}=\lambda_{D,\mathrm{eff}}+r_{e}$$

Equation (21)–(23) give a very crude approximation of the influence of the double layer on the parameter $\alpha_{i}$ . However, more advanced models, which are able to describe the exponential potential decay in the double layer, require to solve an additional system of differential equations.[^61^, ^62^, ^63^, ^64^] This would result in more time‐intensive calculations. Since the aim of our model is to describe qualitative trends relative to G1 the approximation discussed above provides a sufficient estimate of the influence of the solvent on double layer properties. Therefore, the simple approach via a linear potential decay in the double layer [Eqs. (21)–(23)] is adequate for this study and a revised model taking into account double layer properties in more detail will be derived in future work.

## Results and Discussion

### Magnesium deposition mechanism

An open question for the magnesium deposition mechanism is how many solvent molecules need to be stripped from the fully solvated magnesium cation before it can be reduced at the electrode surface. This is a crucial issue since the structure of the (partially) desolvated intermediate is the basis for determining the main parameters of the kinetic model ($K_{\mathrm{desol}}^{0}$ and $\alpha_{i}$ ). DFT calculations and experimental measurements provide a first insight on how much desolvation is required prior to the electron transfer.

**Figure 3:**
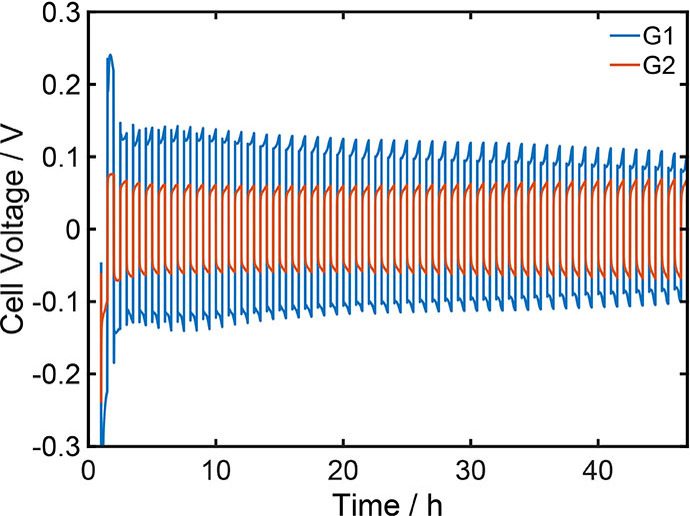
Cycling of symmetric magnesium coin cells with 0.3 M Mg[(hfip)_4_]_2_ electrolyte based on DME and G2 at a current density of 1 mA cm^−1^.

It was observed, that the use of G2 instead of G1 significantly reduces the overpotential during cycling of symmetric magnesium cells (Figure 3). Due to its higher viscosity the transport in G2 is slower than in G1 (Table 1). This leads to a larger concentration gradient and higher ohmic losses in the cell, especially at the quite high current density of 1 mA cm^−2^, at which the symmetric cell was operated (Figure 3). Both effects should cause higher overpotentials in the cell. However, the experimental results (Figure 3) strongly suggest, that the magnesium deposition and/or dissolution is significantly faster in G2 than in G1. Thereby, it can be assumed, that the main influence of the solvent is related to the plating reaction, during which the solvation shell of the magnesium cation has to be removed.

**Figure 4:**
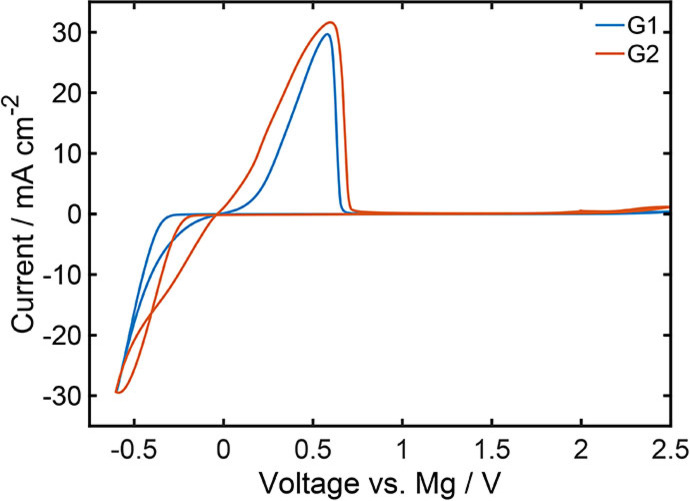
Cyclic voltammograms in 0.3 M Mg[(hfip)_4_]_2_ electrolyte based on DME and G2 (10th cycle) using Pt as working and Mg as counter electrode at a scan rate of 50 mV s^−1^.

This assumption is confirmed by cyclic voltammetry (CV) (Figure 4). After conditioning, during which the overpotential for magnesium plating decreases and the current density increases (Figure S1),^[25]^ it can be clearly seen, that the oxidation peak, which is caused by magnesium stripping, occurs at similar overpotentials for G1 and G2. Consequently, the solvent seems not to have a significant impact on the magnesium dissolution, which is an important finding for describing the stripping kinetics [Eqs. (3) and (14)]. In contrast the reductive peaks associated with magnesium plating seem to appear at different overpotentials in G1 and G2. Thereby, the reductive peak current in G2 appears at a similar overpotential as the oxidative one, whereas the reductive peak in G1 is cut off and therefore occurs at a higher overpotential (Figure 4). Consequently, G2 seems to significantly facilitate the magnesium deposition compared to G1. Most probable reason is an easier desolvation of the electrochemically active specie which will be discussed in the next paragraph.

Since the dielectric constant and the size of the solvated magnesium cations are quite similar in G1 and G2 (Table 1), the desolvation energy $\Delta G_{\mathrm{desol}}$ will mainly be responsible for the different rates of the desolvation reaction [Eqs. (12) and (20)] and therefore for the different overpotentials (Figure 3). Consequently, the energy, which is required for the desolvation of a G2‐solvated magnesium cation, should be significantly smaller than for a G1‐solvated one (Δ*G*_desol (G2)_≪Δ*G*_desol (G1)_).

**Figure 5:**
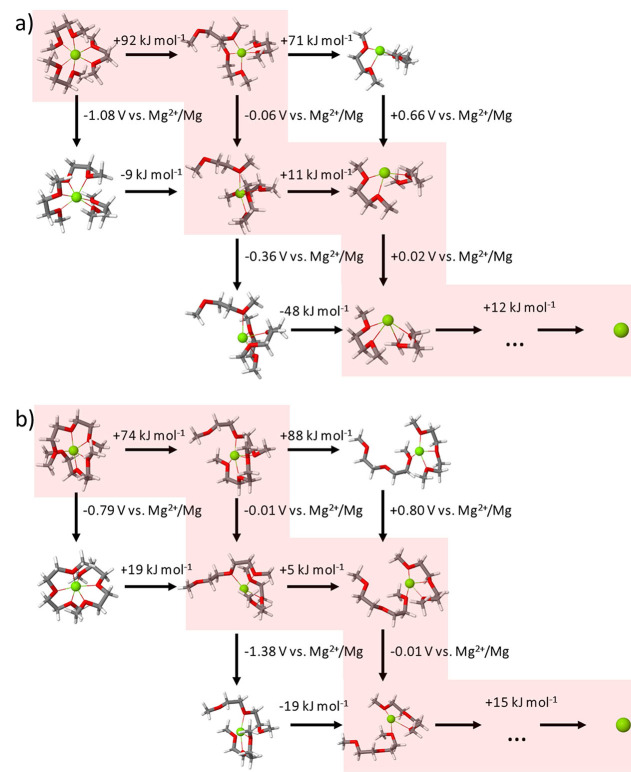
Analysis of possible desolvation (horizontal) and reduction (vertical) processes for a) Mg(G1)${}_{3}^{2+}$ and b) Mg(G2)${}_{2}^{2+}$ .

With this finding DFT calculations can give an important hint about processes that happen at the magnesium electrode. As described above the deposition of the metallic magnesium from the electrolyte is mainly determined by two different processes: desolvation and electron transfer. The difficulty of modeling of the entire process on the atomistic scale is the overlap of both reactions, dynamic competition, and even their interdependence. It is intuitive that desolvation is a bottleneck of the electrode process – electron transfer is usually much faster than any acid‐base reaction^[65]^ – and reduction will occur the moment that the magnesium cation approaches the electrode surface, with a thermodynamic reduction potential equal or above the electrode potential, i. e. 0 V vs. Mg^2+^/Mg. That renders the determination of reaction path a two‐dimensional problem, as shown in Figure 5, with desolvation reactions displayed horizontally and reduction shown along the vertical axis.

It becomes clear that reduction of fully solvated species is impossible both in case of G1 and G2, indicated by values far below 0 V vs. Mg^2+^/Mg. A high level of cation solvation inhibits electron transfer to the magnesium cation, as there is no space around Mg^2+^ to locate an additional electron. However, desolvation of a single coordination site already brings the E_red_ values very close to the potential of magnesium electrodes, resulting in reduction potentials of −0.06 and −0.01 V vs. Mg^2+^/Mg for electroactive species with G1 and G2 as a solvent, respectively. That indicates that the transfer of one electron happens just after desolvation of a single coordination site, reducing the charge of the cation and facilitating further desolvation – the corresponding energy needed for desolvation decreases significantly. Figure 5 clearly shows that the energies of the initial desolvation is few times higher than for the ones following reduction. Consequently, the desolvation of one of the six coordination sites is the most crucial step and determines the kinetics of the entire plating process. After the initial desolvation and the first electron transfer, the further deposition pathway is going through desolvation of the second coordination site which is required for the transfer of the second electron. Finally, the remaining solvent molecules still bound to Mg^0^ are removed. The entire predicted pathway of elementary steps of the electrode reactions are marked with red in Figure 5, displaying huge similarity between G1‐ and G2‐based systems. That allows us to generalize the conclusions of this detailed analysis of the magnesium deposition to other solvents. The study indicates that just the initial desolvation reaction is determining the kinetic description of the deposition process [Eqs. (4) and (12)].

To validate that finding, we go back to the experimental observations (Figure 3). The calculated first desolvation energies correlate well with observed differences of the overpotentials: Energies of 92 and 74 kJ mol^−1^ are required to create the first free coordination site in Mg(G1)${}_{3}^{2+}$ and Mg(G2)${}_{2}^{2+}$ complexes respectively, explaining the noticeable decrease in voltage required to cycle symmetric magnesium cells after substitution of G1 with G2. An additional study of the relative rates for desolvation in G1 and G2 on the basis of the complete kinetic model [Eq. (S19)] also indicates that the initial desolvation step is determining the total deposition rate (Figure S2).

Finally, it should be mentioned that the DFT calculations were performed in a bulk implicit solvent, neglecting the presence of the electrode. The evaluated thermodynamic conditions for electron transfer are critical, but only if a short distance between the electrode and magnesium cation is assumed. The spatial proximity of the magnesium cation to the electrode surface is also a crucial factor for the deposition, since the probability for electron tunneling decreases exponentially with the distance between the electron donor and acceptor. Thus, desolvation not only provides thermodynamical ability to accept electrons, but also enables the magnesium cation to get close enough to the electrode surface for its reduction. Thereby, a physical or chemical bond might be formed between the magnesium cation and the negatively charged electrode surface, so that its typical coordination number of six can be restored.

All in all, DFT calculations and the measurements with G1 and G2 (Figure 3 and 4) strongly indicate that it is reasonable to assume desolvation as rate‐determining step for the deposition reaction [Eqs. (4) and (12)]. Additionally, the DFT study shows that the relevant intermediate i of the kinetic model has one Mg−O bond less (CN=5) compared to the fully solvated cation (CN=6). Interestingly, in the case of glymes, which are multidentate solvents, the rate‐determining initial desolvation (Figure 5), which enables the first electron transfer, does not include the loss of a solvent molecule (w=0 ). The DFT calcuations also provide insights about the structure and the thermodynamic stability of this intermediate, which is the basis for determining the parameters of the kinetic model on the continuum scale [Eqs. (17)–(23)]. The resulting values are summarized in Table 2.

**Table 2:** Kinetic parameters for Mg^2+^ reduction in different solvents.

Parameter	G1	G2	G3	G4	THF
*w*	0	0	0	0	1
*r*_i_ [Å]	5.549	5.855	7.854	7.694	5.568
$\Delta G_{\mathrm{desol}}$ [kJ mol^−1^]	92	74	90	85	76
$k_{\mathrm{desol}(\mathrm{Sol})}^{0}/k_{\mathrm{desol}(G1)}^{0}$	1.0	1423.9	2.2	16.8	635.4
$c_{i,\max(\mathrm{Sol})}/c_{i,\max(G1)}$	1.0	0.898	0.499	0.520	0.993
$K_{\mathrm{desol}(\mathrm{Sol})}^{0}/K_{\mathrm{desol}(G1)}^{0}$	1.0	1278.5	1.1	8.7	631.0
$\lambda_{D,\mathrm{eff}}$ [Å]	3.349	3.432	3.477	2.614	3.415
*α*_i (0.2 M)_	0.283	0.256	0.109	0.177	0.387

### Influence of the double layer on desolvation

The parameter $\alpha_{i}$ of the kinetic model can be interpreted as a measurement to what extent the potential decay in the electrochemical double layer supports the desolvation reaction. This is important since the desolvation has to happen quite close to the electrode surface to enable magnesium deposition. In general, a larger value for $\alpha_{i}$ implies that the electrochemical double layer promotes desolvation. All in all, the influence of the electrochemical double layer on the kinetics is very complex even though a very simple approach is used to determine the corresponding parameter $\alpha_{i}$ [Eq. (21)–(23)]. More details can be found in the Supporting Information (Section S2.2).

Since our simple approach to determine $\alpha_{i}$ [Eqs. (21)–(23)] leads to an unrealistically strong concentration dependence (Figure S3) the simulations for the symmetric magnesium cells are done for the two extreme cases: On the one hand, a concentration‐dependent $\alpha_{i}$ is considered, whereby *α_i_* is set to zero as soon as the model predicts negative values and corresponding results are indicated. On the other hand the constant value for the electrolyte concentration of 0.2 M is used (Table 2). With this method it is possible to get a better insight on the variation range of the results.

### Rate constants of the desolvation and oxidation reactions

**Figure 6:**
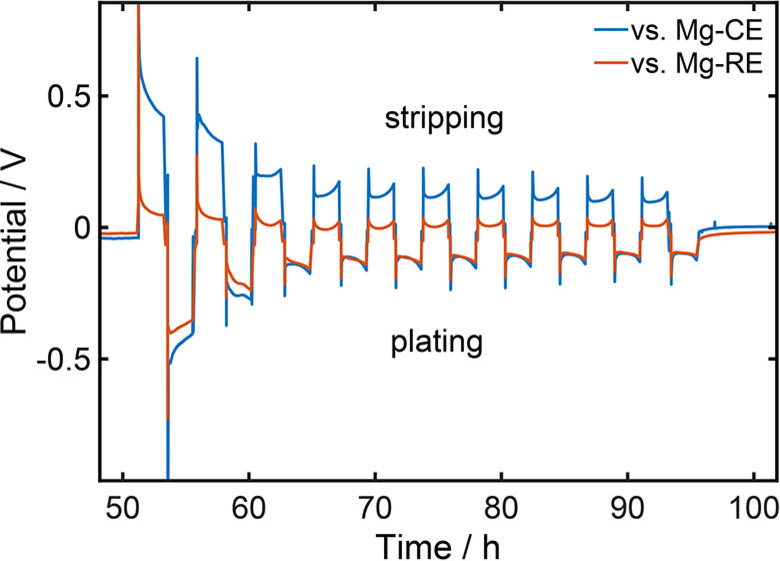
Cell and half cell voltage of a symmetric magnesium cell and a 0.2 M Mg[(hfip)_4_]_2_/G1 electrolyte at a current density of 0.1 mA cm^−1^.

A magnesium reference electrode enables to distinguish between the contributions of the plating and stripping reaction to the overall cell voltage. For the commonly used solvent G1 it can be seen, that the overpotentials for magnesium deposition is significantly higher compared to the overpotential for metal dissolution (Figure 6). Consistent with the CV measurements and the DFT data (Figure 4 and 5), this experimental observation strongly indicates that the desolvation of the magnesium cation hinders the plating reaction.

Note, that surface energetics related to nucleation can also cause an asymmetry between deposition and dissolotion.^[66]^ However, measurements with different solvents show, that the plating and stripping overpotentials can be almost similar. Therefore, the asymmetry is mainly caused by solvent effects and the influence of the nucleation can be neglected. Consequently, the ratio between the overpotentials during magnesium depositon and dissolution can be used to get insights in the rate constants for the desolvation and oxidation reaction. During cycling the surface morphology of the electrodes changes, which leads to an exponential decrease of the overpotential with the number of cycles (Figure 6).^[26]^ This also causes the reference potential and therefore the open‐circuit voltage (OCV) to slightly shift during cycling. For that reason, the values for the overpotentials are corrected by the OCV measured directly before the direction of the electric current was changed (Figure S9). A more detailed discussion on the evaluation of the experimental data can be found in the Supporting Information (Section S3.1).

**Figure 7:**
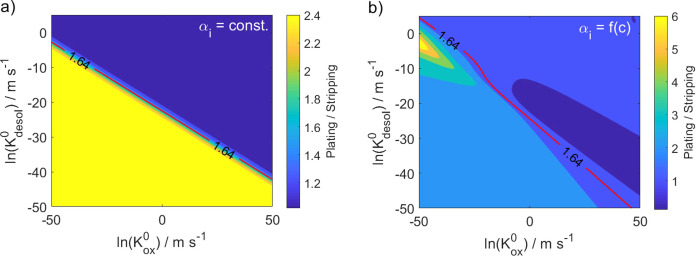
Parameter study: Influence of the rate constants $K_{\mathrm{desol}}^{0}$ and $K_{\mathrm{ox}}^{0}$ on the ratio between the overpotentials of plating and stripping for a 0.2 M Mg[B(hfip)_4_]_2_/G1 electrolyte, a current density of 0.1 mA cm^−1^ and a constant (a)/concentration‐dependent (b) value for $\alpha_{i}$ . The red line indicates the experimentally observed value of 1.64 (Table 4).

Analysis of our experimental data provides a ratio between magnesium plating and stripping in G1 of around 1.64 at a low current density of 0.1 mA cm^−2^ (Table 4). With this in mind a parameter study is done, in which different values for the two rate constants $K_{\mathrm{desol}}^{0}$ and $K_{\mathrm{ox}}^{0}$ are investigated (Figure 7). By evaluating their impact on the asymmetry of the overpotentials a relation between the two rate constants is found, which can represent the experimental observations. For a constant $\alpha_{i}$ a perfectly linear relation is found between the logarithm of the oxidation and desolvation rate constant (Figure 7a). For a concentration‐dependent $\alpha_{i}$ (Figure 7b) the results are more complex. Still, in both cases the desolvation rate constant needs to decrease with an increasing oxidation rate constant. Thereby, the decrease is more pronounced, when the concentration‐dependence of $\alpha_{i}$ is considered.

Knowing the ratio of the rate constants determining the asymmetry of the overpotentials for magnesium deposition and dissolution (Figure 7), the overall cell voltage can be used as additional side condition to determine the magnitude of the rate constants $K_{\mathrm{ox}}^{0}$ and $K_{\mathrm{desol}}^{0}$ (Figure S4). Thereby, the magnitude of the cell voltage is almost identical for a constant and a concentration‐dependent $\alpha_{i}$ . Surprisingly, in the case of the constant $\alpha_{i}$ the experimentally observed value for the cell voltage is reproduced well by the simulation for all $K_{\mathrm{ox}}^{0}$ and $K_{\mathrm{desol}}^{0}$ value pairs of the linear relation, which describe a ratio between the half cell potentials of 1.64 (Figure S4a). Consequently, it is not possible to determine an unique set of parameters for the rate constants when $\alpha_{i}$ is considered as a constant. Therefore, following simulations are done for multiple value pairs of the two rate constants ($-50<\ln\left(K_{\mathrm{ox}}^{0}\right)<50$ ) and the mean as well as the standard deviation are determined. However, the good agreement of both, the asymmetry of the half cell potentials and the overall cell voltage (Figure 7a and S4a), indicates, that the simple model for the electrochemical double layer [Eqs. (21)–(23)] predicts a very reasonable value for the parameter $\alpha_{i}$ .

In contrast, there is no direct correlation between the cell voltage and the ratio of the half cell potentials, when the concentration‐dependence of $\alpha_{i}$ is considered (Figure 7b and S4b). Consequently, in this case there is only one value pair of the two rate constants, for which the simulation predicts the experimentally observed cell voltage. A rough estimation for the two rate constants in G1 can be given (Table 3) by including the standard deviation (sdv) of the experimentally determined values for the asymmetry of the overpotentials as well as for the cell voltage (Table 4). It can be seen, that the rate constant for the desolvation and therefore for the entire deposition reaction seems to be significantly smaller than the one for the oxidation. Since it is assumed, that the desolvation is slower than the electron transfer, this finding could be expected. Figure 7 implies, that the concentration dependence of $\alpha_{i}$ has a significant impact on the simulations results. However, when the desolvation rate constant $K_{\mathrm{desol}}^{0}$ is determined for $\alpha_{i}=const.$ on the basis of the estimated value for the oxidation rate constant $K_{\mathrm{ox}}^{0}$ (Table 3, $\alpha_{i}=f\left(c\right)$ ), it can be seen, that its value ($2.01\cdot 10^{-8}$ m s^−1^) is very close to the value of $K_{\mathrm{desol}}^{0}$ , when the concentration dependence of $\alpha_{i}$ is considered ($2.00\cdot 10^{-8}$ m s^−1^). Since the concentration gradients in G1 are very small at the analyzed current density of 0.1 mA cm^−2^ (Figure S7), this result is not surprising.

**Table 3:** Rate constants for magnesium oxidation and desolvation in Mg[(hfip)_4_]_2_/G1 for $\alpha_{i}=f\left(c\right)$ .

$K_{\mathrm{ox}}^{0}\left[{\mathrm{ms}}^{-1}\right]$		$K_{\mathrm{desol}}^{0}\left[{\mathrm{ms}}^{-1}\right]$	

mean	sdv	mean	sdv
1.7 ⋅ 10^−5^	2.1 ⋅ 10^−5^	2.0 ⋅ 10^−8^	1.7 ⋅ 10^−8^

**Table 4:** Cell voltage, its asymmetry, and corresponding standard deviation (sdv) for 0.2 M Mg(B[hfip]_4_)_2_ in different solvents at a current density of 0.1 mA cm^−2^.

Solvent	Plating/Stripping		Cell voltage [V]	
	mean	sdv	mean	sdv
G1	1.64	0.07	0.067	0.016
G2	1.15	0.04	0.041	0.013
G4	2.38	0.64	0.124	0.034
THF	0.95	0.03	0.043	0.011

The determined rate constant for the oxidation reaction can be applied for all solvents, whereas the corresponding desolvation rate constant has to be adjusted for the different solvents following Eq. (17) and (20). The final set of parameters is listed in Table 2.

## Experimental validation

### Influence of the solvent

**Figure 8:**
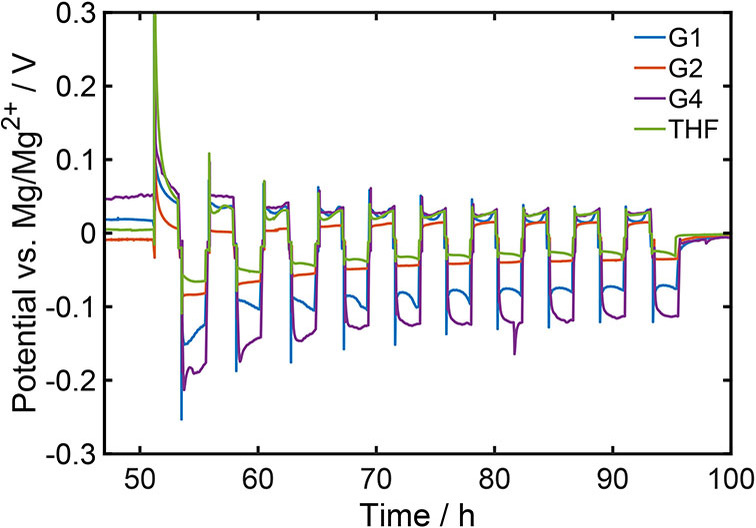
Cycling of symmetric magnesium cells with a magnesium reference electrode and a 0.2 M Mg[(hfip)_4_]_2_ electrolyte based on different solvents at a current density of 0.1 mA cm^−1^.

Figure 8 and S5 show the potential against a magnesium reference electrode, which was measured during the cycling of a symmetric magnesium cell with 0.2 M Mg[B(hfip)_4_]_2_ electrolyte based on different solvents at a current density of 0.1 mA cm^−1^. The corresponding values for the ratio between the overpotentials during plating and stripping as well as the ones for the cell voltage are summarized in Table 4.

**Figure 9:**
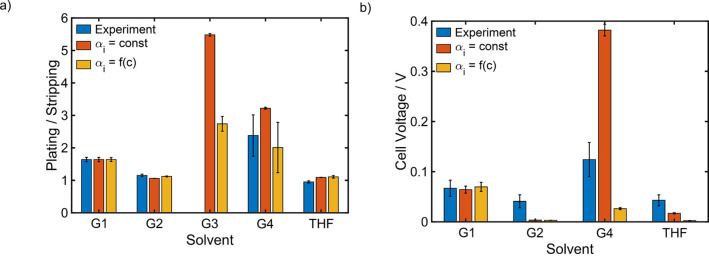
Comparison between experimental data and simulation results for the asymmetry of the overpotentials during plating and stripping (a) and the cell voltage (b) of symmetric magnesium cells with a 0.2 M Mg[B(hfip)_4_]_2_ electrolyte based on different solvents at a current density of 0.1 mA cm^−1^.

It can be seen, that the overpotential during magnesium dissolution is very similar for all solvents (Figure 8 and S5), which was already observed by CV measurements (Figure 4). This supports the assumption, that the rate of the stripping is independent of the solvent [Eqs. (3) and (14)]. In contrast, during magnesium deposition there are significant differences in the overpotentials between solvents investigated in this work. This behavior might be ascribed to the desolvation of the magnesium cation. Thereby, a lower desolvation energy should lead to a smaller overpotential during magnesium plating and, therfore, plating/stripping ratios close to unity. Consequently, in G2 and THF the desolvation energy for the magnesium cation should be the lowest, whereas it should be the highest in G4. For the relevant desolvation of the first Mg−O bond DFT calculations predict, that the required energy is the lowest for G2 (Table 2). Moreover, for THF the calculated desolvation energy is only slightly higher. These results perfectly fit to the experimental observations and indicate, that in these cases the thermodynamics of the rate‐determining initial desolvation step is mainly responsible for the plating overpotential. However, the DFT calculations predict the highest desolvation energy for G1 (Table 2) and not for G4, as it is suggested by the experiments (Figure 9 and S5). This shows, that the impact of the solvent on the battery performance is more complex and the desolvation energy is not sufficient as a sole descriptor. Apart from the thermodynamics of the desolvation, the solvent also affects the ion transport in the bulk electrolyte as well as the properties in the electrochemical double layer close to the electrode surface.

Consequently, the desolvation energy is only a good descriptor for the battery performance, when transport limitations, steric effects and the dielectric constant of the solvents are very similar. Otherwise, a more complex model is required, which takes into account all the different aspects of the solvent properties. With the kinetic model [Eq. (15)] developed in this work we provide a simple yet efficient approach to explicitly include the desolvation as well as the impact of the electrochemical double layer. Additionally we take into account transport effects by consistently coupling it to a general transport theory [Eqs. (S8)–(S10)].

Figure 9 compares simulation results for a constant as well as a concentration dependent $\alpha_{i}$ to experimental data. Since the ratio between the half cell potentials in G1 was used as input for the determination of model parameters, the values of the simulation and the experiment are the same (Figure 9a). The same applies to the cell voltage for the simulation with a concentration dependent $\alpha_{i}$ . In contrast the cell voltage for constant $\alpha_{i}$ is a direct consequence of the ratio between the overpotentials during plating and stripping (Figure S4a). Its good agreement with the experimental result suggests, that the determined value for $\alpha_{i}$ provides a fairly good estimation. For G2 and THF the ratio of the half cell potentials is very close to one, no matter whether the concentration dependence of $\alpha_{i}$ is considered or not (Figure 9a). This does not only fit perfectly to the experimental data, but it also shows, that there are no significant transport limitations in these two solvents, at least at the low current density of 0.1 mA cm^−2^, which could also be seen in the simulations (Figure S7). Moreover, the dielectric constant of G2 and THF (Table 1) as well as the radii of the partially desolvated intermediate (Table 2) are quite close to the ones of G1. Therefore, the simple comparison of the desolvation energies could already give a good picture about the electrochemical performance in those three solvents.

In contrast, simulation results for the two different cases of $\alpha_{i}$ differ significantly for G4 (Figure 9). This indicates, that the transport in this solvent is considerably slower than in the other ones, which can also be seen by the corresponding transport parameters (Table 1). Therefore, the concentration gradient, which build up during battery cycling (Figure S7), is not negligible anymore, even at the low current density of 0.1 mA cm^−2^. Consequently, magnesium depletes at the electrode, where the plating takes place. On the one hand, a smaller concentration is unfavorable for the kinetics [Eq. (12)]. But on the other hand, a depletion of magnesium leads to an increase of $\alpha_{i}$ (Figure S3), which in turn favors the desolvation reaction. Thereby, the reaction rate for the desolvation is exponentially dependent on $\alpha_{i}$ , but only linearly dependent on the concentration of the active specie [Eq. (12)]. Consequently, the transport limitation seems to be favorable for the desolvation especially for higher potential differences between electrode and electrolyte. This effect leads to a considerably lower cell voltage and a lower ratio of the half cell potentials, when the concentration dependence of $\alpha_{i}$ is considered. For that reason, simulations with constant $\alpha_{i}$ overestimate both, the cell voltage and the ratio of the two half cell potentials. Note, that as discussed above our simple model for the electrochemical double layer overestimates the concentration dependence of $\alpha_{i}$ (Figure S3). Therefore, the simulation result for a concentration dependent $\alpha_{i}$ underestimate the overpotentials. As a result, the experimental values should be somewhere in between of the two simulation results, which is indeed the case for G4 (Figure 9) where concentration gradients are most pronounced (Figure S7).

All in all, the results for G4 are the most interesting ones. Although the desolvation energy is lower than for G1 (Table 2), which indicates that the desolvation reaction should be more than 16 times faster in G4, the experimental observation shows significantly higher overpotentials compared to G1. Consequently, other solvent effects destroy this thermodynamic advantage of G4. Figure 9 shows, that the full cell model can reproduce the trend of the experimental observation. Thereby, it seems that the effect of the electrochemical double layer is mainly responsible for the slower desolvation kinetics. Especially the large size of the partially desolvated intermediate seems to play an important role. In general, steric effects can considerably affect the desolvation kinetics. Since G4 is a sterically very demanding solvent and it can be assumed, that the magnesium cation needs to get quite close to the electrode surface, the desolvation of one coordination site might be not sufficient to enable the magnesium deposition in G4. Therefore, also further desolvated intermediates with two and three free coordination sites were analyzed (Figure S8). Thereby, the coordination number of three is connected to the loss of one solvent molecule before the electron transfer. By considering both, the asymmetry of the half cell potentials and the overall cell voltage, it seems to be most likely, that the desolvation of one coordinating oxygen atom is still sufficient to enable the reduction of the magnesium cation even for the very bulky solvent G4 (Figure S8). Consequently, steric effects seem to be an important factor regarding the influence of the electrochemical double layer on the kinetics as well as regarding the transport via diffusion in the electrolyte [Eq. (16)], but they do not seem to change the mechanism of the magnesium deposition.

Note, that the predicted values for the cell voltage in G2 and THF are extremely small and therefore significantly smaller than the measured ones (Figure 9). Moreover, the overpotentials during plating and stripping in those two solvents are almost equal (Figure 4 and 9). These two findings indicate, that the desolvation is not significantly limiting the magnesium deposition, which would imply, that the electron transfer reaction is mostly independent of the solvent. This assumption is also supported by the fact, that the experimentally observed asymmetry and especially the measured cell voltage are very similar for G2 and THF (Figure 8). Moreover, a parameter study regarding the impact of the desolvation energy (Section S3.4) clearly shows, that the adverse effect of the desolvation on magnesium deposition is minor for energy barriers below 80 kJ mol^−1^ (Figure S16). In these cases the plating/stripping ratio is mainly determined by the transport in the electrolyte. Consequently, the overpotentials during plating and stripping are almost symmetric in G2 and THF due to sufficient small desolvation energies (74 and 76 kJ mol^−1^, Table 2) and no significant transport limitations (Table 1). Since the impact of the desolvation on magnesium deposition is found to be minor in G2 and THF, the interface kintics in these two solvents can also sufficiently be described by the common Butler‐Volmer equation (Eq. (S20) and Figure S15). The corresponding parameters are given in the Supporting Information (Table S7).

To get a complete picture of the commonly used solvents for magnesium batteries G3 is analyzed in addition to the experimentally validated solvents G1, G2, G4 and THF (Figure S6). The simulations clearly indicate, that from all analyzed solvents G3 seems to be the one with the most detrimental effect on the desolvation and, therefore, on the battery performance (Figure 9). Consequently, G3 is in this aspect not a good solvent for magnesium batteries.

All in all, the required partial desolvation of the magnesium cation seems to be easiest in G2 and THF. Consequently, the best battery performance in terms of small overpotentials (Figure 9) can be achieved with these two solvents. However, for THF this is only the case for low current densities (<0.2 mA cm^−1^, Figure S13). At higher current densities the potential difference at the electrode/electrolyte interface seems to be high enough to enable an undesired decomposition of THF molecules,[^67^, ^68^, ^69^] which adversely effects the battery performance. Moreover, for safety reasons THF won't be the solvent of choice anyway, since its boiling/flash point is significantly lower than in the case of G2 (THF: 66 °C/−14 °C and G2: 162 °C/57 °C).^[70]^ Consequently, the most promising ethereal solvent for magnesium batteries is G2.

### Influence of the current density

To further validate our kinetic model, the influence of the current density is analyzed. Thereby, we focus on our reference solvent G1 as well as the most promising candidate G2. Since the change of the overpotentials in G2 at increasing current density is rather small (Figure S10) and the impact of the desolvation was found to be less pronounced (Figure 8) the most reliable results for model validation can be expected for G1. Figure S9 shows the measured overpotentials during cycling of symmetric magnesium cells at four different current densities. The evaluated values for the ratio between the two half cell potentials and the cell voltages are summarized in Table 5.

**Table 5:** Cell voltage and its asymmetry for 0.2 M Mg(B[hfip]_4_)_2_/G1 at different current densities.

Current density	Plating/Stripping		Cell Voltage [V]	
[mA cm^−2^]	mean	sdv	mean	sdv
0.1	1.64	0.07	0.067	0.016
0.2	1.78	0.19	0.083	0.024
0.5	1.62	0.06	0.117	0.033
1.0	1.48	0.11	0.139	0.052

**Figure 10:**
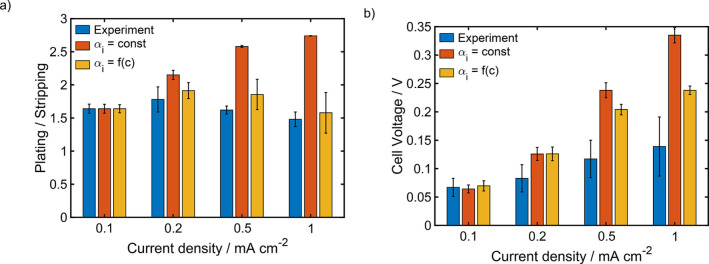
Comparison between experimental data and simulation results for the asymmetry of the overpotentials during plating and stripping (a) and the cell voltage (b) of symmetric magnesium cells with a 0.2 M Mg[B(hfip)_4_]_2_/G1 electrolyte at different current densities.

As expected, the overall cell voltage increases with increasing current density (Table 5). This feature can be captured by the model, whereby it doesn't matter, whether the concentration dependence of the double layer parameter $\alpha_{i}$ is considered or not (Figure 10). More interesting is the behavior of the ratio between the overpotentials during plating and stripping. After a slight increase the experimentally measured value decreases with increasing current density (Table 5 and Figure 10). In contrast the simulations with a constant $\alpha_{i}$ predict a very pronounced increase of the ratio between the half cell potentials. However, this result is not surprising, since a strong correlation between the cell voltage and the ratio of the half cell potentials was already observed before for a constant $\alpha_{i}$ (Figure 7, 9 and S4). Interestingly, simulations with concentration dependent $\alpha_{i}$ exactly capture the unexpected behavior of the experimentally observed plating/stripping ratio (Figure 10). This result indicates, that the very simple model to determine $\alpha_{i}$ is able to qualitatively describe the influence of the concentration at the electrode surface. This is also the case for G2 (Figure S11). In contrast to G1, in G2 both, the cell voltage and the ratio between the half cell potentials, slightly increase with an increasing current density (Table S5).

### Modeling of electron‐transfer kinetics in magnesium electrolytes

The comparison to the commonly applied Butler‐Volmer approach (cf. Section S3.3) clearly shows the advantages of the herein presented kinetic model. All in all, the Butler‐Volmer equation [Eq. (S20)] is often adequate to describe experimentally measured data. However, it is of phenomenological nature and therefore can not be used to predict the influence of the desolvation on the battery performance, which is needed to screen new solvents for magnesium batteries. Moreover, for complex systems like magnesium electrolytes many different aspects can play an important role for the electron‐transfer kinetics, e. g. desolvation, the electrochemical double layer as well as multiple active species. For such systems the simple Butler‐Volmer approach reaches its limits, whereas all those aspects can easily be considered in the herein presented model. This gets even more important for describing magnesium intercalation cathodes since a full desolvation is required before the magnesium cation can intercalate into the active material. We found that already the initial desolvation of one coordination site can crucially hinder the electron‐transfer reaction in magnesium electrolytes (Figure 5). Therefore, it has to be assumed that the impact of the desolvation on the intercalation kinetics is even more severe. Consequently, the desolvation should explicitly be considered kinetic models of Mg‐ion batteries.

### Influence of the solvent on the battery performance

In general the solvent determines the thermodynamics of the desolvation, the transport in the electrolyte and the electrochemical double layer, which all together impact the magnesium deposition and dissolution kinetics and, therefore, the battery performance. Since a higher current density leads to larger fluctuations of magnesium cations at the electrode surface, the transport in the electrolyte and, therefore, even small differences of the transport properties in the different solvents become more important. Moreover, a higher current density also results in a higher potential difference at the electrode electrolyte interface, which is connected to a more pronounced influence of $\alpha_{i}$ [Eq. (12)] stemming from the structure of the electrochemical double layer. Consequently, the different solvents and current densities can have a very complex influence on the battery performance. However, our kinetic model, which considers the required desolvation of the magnesium cations as well as the impact of the electrochemical double layer, is able to qualitatively reproduce the complex behaviour in the different solvents (Figure 9, 10 and S11).

Overall, the comparison between the experimental data and the simulation results clearly shows (Figure 9, 10), that not only the thermodynamics of the magnesium desolvation but also the electrochemical double layer has an important influence on magnesium electrochemistry and dissolution kinetics. Finally, it was found, that key to low magnesium plating overpotentials are a desolvation barrier <80 kJ mol^−1^ as well as a fast transport in the electrolyte (Figure S16).

## Conclusions

In summary, our combination of different modelling techniques with experimental measurements provides more insights on the influence of the solvent on the mechanism and rate limiting steps for magnesium plating and stripping. Detailed understanding of this mechanism is key to improve the performance of rechargable magnesium batteries. We found that magnesium dissolution is independent of the solvent and the desolvation of the magnesium cation is limiting the deposition rate in chloride‐free electrolytes. Thereby, even for bulky solvents only one coordination site of the solvated magnesium cation needs to be desolvated before the first electron transfer takes place. After a second Mg−O bond is broken the magnesium cation accepts the second electron before remaining solvent molecules are stripped. Thereby, the initial desolvation from CN=6 to CN=5 requires the highest energy and therefore determines the plating reaction. Among the five analyzed ethereal solvents diglyme and THF are the ones, in which the desolvation hinders the magnesium deposition the least. However, when taking into account additional properties, such as stability or low volatility and flammability, diglyme clearly is the most promising solvent for magnesium batteries.

Moreover, a new general kinetic model was developed, which not only considers the required (partial) desolvation of the electroactive cation but also the influence of the electrochemical double layer on the deposition and dissolution kinetics. Both effects were observed to be equally important to reproduce the experimentally observed influence of different solvents and current densities. Although a very simple approach is chosen to describe the electrochemical double layer, the simulations can qualitatively capture all trends in the experimental data.

The analysis in this work focuses on the performance of magnesium metal half cells as well as symmetric magnesium cells, which are of limited practical relevance. However, the desolvation will be important for cathode materials as well, especially for intercalation electrodes. Therefore, our proposed kinetic model can be readily extended also to full cell simulations.

Further improvements of the model and its parametrization should set a focus on the description of the electrochemical double layer which was found to have a significant effect on the deposition reaction. Thereby, the challenge is to find a more advanced description for the electrochemical double layer without significantly increasing the computational effort. Moreover, the kinetic model should be transferred to chlorine containing electrolytes as well as to intercalation materials.

## Conflict of interest

The authors declare no conflict of interest.

## Supporting information

As a service to our authors and readers, this journal provides supporting information supplied by the authors. Such materials are peer reviewed and may be re‐organized for online delivery, but are not copy‐edited or typeset. Technical support issues arising from supporting information (other than missing files) should be addressed to the authors.

Supporting Information

Supporting Information
