# Supplementary material for: Modeling of Electron‐Transfer Kinetics in Magnesium Electrolytes: Influence of the Solvent on the Battery Performance
Source: ChemSusChem. 2021 Oct 7;14(21):4820–35. doi: 10.1002/cssc.202101498 (PMC8597058; doi:10.1002/cssc.202101498)
Supplement: Supplementary file 1 — Supporting Information [file CSSC-14-4820-s001.pdf]

ChemSusChem

Supporting Information

Modeling of Electron-Transfer Kinetics in Magnesium Electrolytes: Influence of the Solvent on the Battery Performance

Janina Drews,* Piotr Jankowski, Joachim Häcker, Zhenyou Li, Timo Danner, Juan Maria García Lastra, Tejs Vegge, Norbert Wagner, K. Andreas Friedrich, Zhirong Zhao-Karger, Maximilian Fichtner, and Arnulf Latz© 2021 The Authors. ChemSusChem published by Wiley-VCH GmbH. This is an open access article under the terms of the Creative Commons Attribution License, which permits use, distribution and reproduction in any medium, provided the original work is properly cited.

S1 Details of the model

S1.1 Modified Davies equation [1]

$$\ln \gamma_{\mathrm{j}} = -\frac{A z_{\mathrm{j}}^2 \sqrt{I}}{1 + r_{\mathrm{j}} B \sqrt{I}} + \frac{C_{(\mathrm{I})} A z_{\mathrm{j}}^2 I}{\sqrt{1000}} \tag{S1}$$

$$A = \frac{\sqrt{2} F^2 e_0}{8\pi (\varepsilon_{\mathrm{r}} \varepsilon_0 R T)^{3/2}} \tag{S2}$$

$$B = \sqrt{\frac{2F^2}{\varepsilon_{\mathrm{r}} \varepsilon_0 R T}} \tag{S3}$$

$$C_{(I)} = -3{,}33 \cdot 10^{-5} \cdot I + 0{,}2 \tag{S4}$$

With the modified Davies equation, which is an extention of the Debye-Hückel model, activity coefficients γ of concentrated electrolytes with ionic strengths $I = \frac{1}{2} \cdot \sum_j z_{\mathrm{j}}^2 c_{\mathrm{j}}$ up to $1500\,\frac{\mathrm{mmol}}{\mathrm{L}}$ [1] can be estimated. Thereby, only the charge number z_{j} and the hydrodynamic radius r_{j} of the ion j, as well as the dielectric constant of the solvent ε_{r} and the temperature T are needed in addition to the fundamental physical constants, which are the Faraday constant F, the elementary charge e_0, the vacuum permittivity ε_0 and the ideal gas constant R.

S1.2 Transport theory [2]

S1.2.1 Transport equations

For a fully dissociated simple magnesium salt MgA_2 like $Mg[B(hfip)_4]_2$ the transport in the electrolyte can be described by the concentration of the salt $c_\pm$. This is true for electrolyte concentrations, at which ion pairing and clustering can be neglected. In this case, the electrolyte concentration $c_\pm$ is directly related to the concentration of solvated magnesium cations c_+, the corresponding anions c_- and the concentration of free solvent c_{Sol}.

$$c_+ = c_\pm \tag{S5}$$

$$c_- = 2c_\pm \tag{S6}$$

$$c_{\mathrm{Sol}} = c_{\mathrm{Sol}}^0 - x c_\pm - 2y - c_\pm \tag{S7}$$

where $c_{\mathrm{Sol}}^0 = \frac{\rho_{\mathrm{Sol}}}{M_{\mathrm{Sol}}}$ denotes the total concentration of free and bound solvent, which is determined by the density ρ_{Sol} and the molar mass M_{Sol} of the pure solvent, and x and y are the solvation numbers of the magnesium cation and the corresponding anion. The conservation of charge as well as the conservation of mass, which is the basis for the relations between the salt concentration $c_\pm$ and the concentrations of the different electrolyte species [Eqs. (S5)-(S7)], enables to formulate the transport theory only in terms of the electrolyte concentration $c_\pm$. In the electrolyte charge conservation leads to the transport equation for the ionic current $\vec{j}_{\mathrm{e}}$:

$$0 = -\vec{\nabla} \vec{j}_{\mathrm{e}} = \vec{\nabla}(\kappa \vec{\nabla} \Phi_{\mathrm{e}}) + \vec{\nabla} \left(\kappa \frac{t_+}{z_+ F} \left(\frac{\partial \mu}{\partial c_\pm} \right) \vec{\nabla} c_\pm \right) \tag{S8}$$

where κ denotes the ionic conductivity of the electrolyte, Φ_{e} the electric potential of the electrolyte, t_+ the transference number of the magnesium cations and μ the effective chemical potential.

Mass conservation results in following differential equation for the particle transport $\vec{N}_\mathrm{e}$ in the electrolyte:

$$\frac{\partial c_\pm}{\partial t} = -\vec{\nabla}\vec{N}_\mathrm{e} = \vec{\nabla}(D\vec{\nabla}c_\pm) - \vec{\nabla}\left(\frac{t_+}{z_+F}\vec{j}_\mathrm{e}\right) \tag{S9}$$

where D is the interdiffusion coefficient. Since magnesium is a conversion electrode, there is no transport of ions in the solid phase ($\vec{N}_\mathrm{s} = 0$) and the electric current $\vec{j}_\mathrm{s}$ simplifies to

$$0 = -\vec{\nabla}\vec{j}_\mathrm{s} = \vec{\nabla}(\sigma\vec{\nabla}\Phi_\mathrm{s}) \tag{S10}$$

where σ stands for the electronic conductivity of the magnesium electrode.

S1.2.2 Effective chemical potential and thermodynamic factor

The effective chemical potential μ enables to consider non-ideality effects of the electrolyte. In general, it's derivate $\frac{\partial\mu}{\partial c_\pm}$, which is part of the transport equations [Eq. (S8)] can be written as a function of the thermodynamic factor f_thermo.[3]

$$\frac{\partial\mu}{\partial c_\pm} = \frac{RT}{c_\pm}\cdot f_\mathrm{thermo} \tag{S11}$$

$$f_\mathrm{thermo} = \sum_j \frac{\partial c_\mathrm{j}}{\partial c_\pm}\cdot\frac{\partial \ln\gamma_\mathrm{j}}{\partial \ln c_\pm} + \left(\frac{\partial c_\mathrm{j}}{\partial c_\pm}\right)^2\cdot\frac{c_\pm}{c_\mathrm{j}} \tag{S12}$$

The thermodynamic factor describes all interactions between the existing species j in the electrolyte, which are the solvated cation (+), the solvated anion (-) and the free solvent molecules (Sol) in the case of a fully dissociated simple magnesium salt. By combining Equation (S5)-(S7) and Equation (S12), the thermodynamic factor for a fully dissociated MgA_2 electrolyte can be described by following expression:

$$f_\mathrm{thermo} = \frac{\partial\ln\gamma_+}{\partial\ln c_\pm} + 1 + 2\cdot\frac{\partial\ln\gamma_-}{\partial\ln c_\pm} + 2 - (x+2y)\cdot\frac{\partial\ln\gamma_\mathrm{Sol}}{\partial\ln c_\pm} + (x+2y)^2\cdot\frac{c_\pm}{c^0_\mathrm{Sol} - xc_\pm - 2yc_\pm} \tag{S13}$$

Since only quite low electrolyte concentrations of 0.2 M are analyzed and it is know that the clustering, which is caused by non-ideality effects, becomes only relevant for significantly higher concentrations (>0.35 M)[3], it is assumed that the bulk electrolyte behaves almost ideally. Moreover, at small current densities only very small concentration gradients occur in the battery cell, so that the concentration dependence of the activity coefficient can be neglected. Therefore, the derivatives of the activity coefficients γ_j can be assumed to be zero. Moreover, for $Mg[B(hfip)_4]_2$ the bulky anion is unsolvated ($y = 0$).[3] This simplifies Equation (S13) to:

$$f_\mathrm{thermo} = 3 + x^2\cdot\frac{c_\pm}{c^0_\mathrm{Sol} - xc_\pm} \tag{S14}$$

S1.2.3 Equilibrium state and influence of the reduction

In equilibrium the current density at the electrode-electrolyte interface vanishes (i_{se}=0). With this condition Equation (15) leads to following expression for the half cell potential $\Delta\Phi^{eq} = \Phi_s^{eq} - \Phi_i^{eq}$:

$$\Delta\Phi^{eq} = \frac{RT}{z_+ F} \cdot \ln\left(\frac{K^0_{desol}}{K^0_{ox}} \cdot c_e^{eq}\right) \cdot \frac{1}{(1-\alpha_{\#2})(1-\alpha_i) + \alpha_{\#1}\alpha_i} \tag{S15}$$

The parameters from Table 2 and 3 can be used to calculate the equilibrium half cell potentials for the different solvents [Eq. (S15)], which are summarized in Table S1.

Table S1 Influence of the solvent on the half-cell potentials $\Delta\Phi^{eq}$ [Eq. (S15)] of a 0.2 M $Mg[B(hfip)_4]_2$ electrolyte.

$\Delta\Phi^{eq}$ [V]	G1	G2	G3	G4	THF
mean	-0.023	0.161	-0.020	0.033	0.143
sdv	0.058	0.058	0.058	0.058	0.058

It can be seen, that the solvent significantly influences the half cell potential (Table 4). Interestingly, there is no correlation between the experimentally observed asymmetry between the overpotential during magnesium deposition and dissolution (Figure 8 and Table 4) and the half cell potential in equilibrium (Table S1). On the one hand transport effects are not considered in the calculation of $\Delta\Phi^{eq}$, which was done for a 0.2 M $Mg[B(hfip_4]_2$ solution for all solvents. On the other hand the double layer parameter α_i does not play any role for the assumed case of $\alpha_{\#1} = \alpha_{\#2} = 0.5$ since the last factor of Equation (S15) ($\frac{1}{(1-\alpha_{\#2})(1-\alpha_i)+\alpha_{\#1}\alpha_i}$) simplifies to a constant value of 2. Consequently, the double layer has no impact on the equilibrium half cell potential. In this case $\Delta\Phi^{eq}$ is only determined by the thermodynamics of the initial desolvation of the magnesium cation (K_{desol}/ ΔG_{desol}). However, during battery operation, which causes a non-equilibrium state, the transport in the electrolyte as well as the electrochemical double layer can additionally affect the kinetics of the interface reactions especially regarding the desolvation process. Therefore, the half cell potential in the equilibrium state (Table S1) is not directly related to the overpotential, which is needed for magnesium deposition (Figure 8).

Usually, the half cell potential of an electrochemical reaction can be described by the Nernst equation:

$$\Delta\Phi^{eq} = \Delta\Phi_0^{eq} + \frac{RT}{zF} \cdot \ln\frac{a_{Ox}}{a_{Red}} \tag{S16}$$

with the standard half-cell reduction potential $\Delta\Phi_0^{eq}$. For an ideal 0.2 M magnesium electrolyte, in which the desolvation does not impact the electron transfer reaction, Equation (S16) predicts a half cell potential of 0.068 V. The comparison with Table S1 shows, that the required desolvation of the magnesium cation shifts the half cell potentials to lower values (for G1, G3 and G4). From the experiments and simulations (Figure 8 and 9) it is assumed, that the desolvation is not significantly limiting the magnesium deposition in G2 and THF, which is also supported by the higher value of the half cell potential with respect to the ideal value. Therefore, the reduction of the partially desolvated magnesium cation becomes relevant for its deposition, which is not captured by our simplified kinetic model [Eq. (15)] and would require a more detailed kinetic description of the individual steps of the plating reaction (Figure 5).

The most simplest approach to include the effect of the electron transfer on the magnesium deposition is to define an effective rate constant for the plating reaction K^0_{plat} based on the rate constants

for the desolvation K^0_{desol} as well as reduction reaction K^0_{red}. This would modify Equation (15) into following more general expression:

$$i_{\text{se}} = z_+ F \cdot \left[K^0_{\text{ox}} \cdot \exp\left(\frac{z_+ F}{RT} (1-\alpha_{\#2})(1-\alpha_{\text{i}})(\Phi_{\text{s}} - \Phi_{\text{e}}) \right) - K^0_{\text{plat}} \cdot c_{\text{e}} \cdot \exp\left(-\frac{z_{\text{e}} F}{RT} \alpha_{\#1} \alpha_{\text{i}} (\Phi_{\text{s}} - \Phi_{\text{e}}) \right) \right] \tag{S17}$$

with

$$\frac{1}{K^0_{\text{plat}}} = \frac{1}{K^0_{\text{desol}}} + \frac{1}{K^0_{\text{red}}} \tag{S18}$$

Thereby, Equation (S18) can be interpreted as a serial connection of resistances for desolvation and electron transfer. When the reduction reaction is fast compared to the desolvation ($K^0_{\text{red}} >> K^0_{\text{desol}}$), as it is indicated by the DFT calculations (Figure 5), Equation (S17) simplifies to Equation (15). However, the more general picture requires an additional parameter, namely K^0_{red}. A very crude assumption could be, that the rate constants for the electron transfer reactions are in the same order of magnitude ($K^0_{\text{red}} \approx K^0_{\text{ox}}$). Consequently, the rate for the initial desolvation K^0_{desol} in G2 and THF (Table 2, 3 and Eq. (20)) seems to be quite close to the one for magnesium reduction K^0_{red}. This fits perfectly to the experimental observations (Figure 8), which imply that the magnesium deposition in G2 and THF is not mainly influenced by the desolvation.

S2 Details of the parameterization

S2.1 Deposition mechanism in magnesium batteries

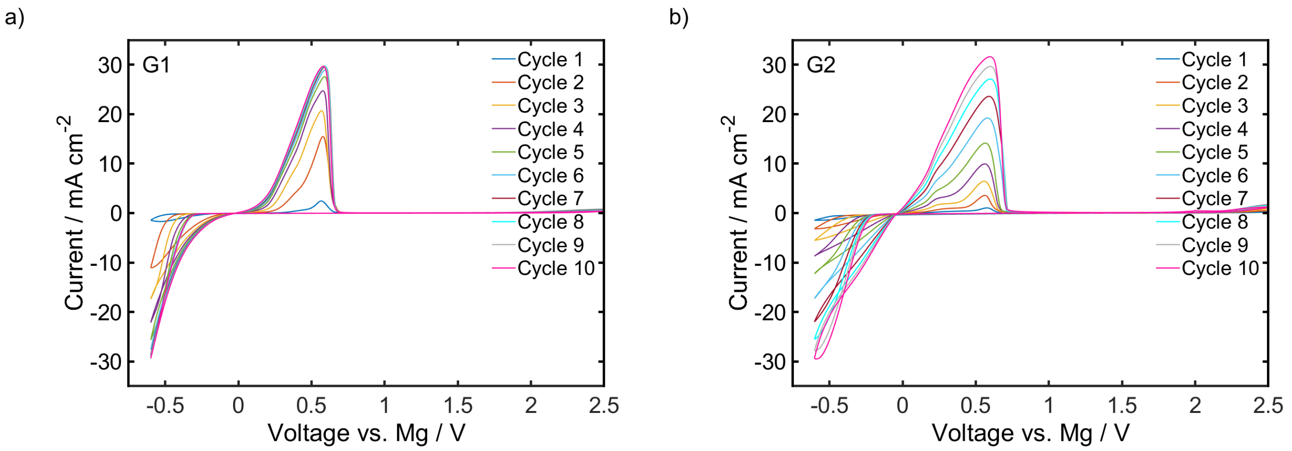

Figure S1 Cyclic voltammograms in 0.3 M $Mg[(hfip)_4]_2$ electrolyte based on DME (a) and G2 (b) using Pt as working and Mg as counter electrode at a scan rate of $50\,mV\,s^{-1}$.

The fundamental question for the parametrization of the kinetic model is, to what extent the magnesium cation has to be desolvated before the electron transfer can take place. In general, this relevant intermediate specie is a magnesium cation, which might be coordinated by 0 - 5 oxygen atoms from the solvent.
When the influence of the different solvents on the transport in the electrolyte can be neglected ($c_{e\,(Sol)} \approx c_{e\,(G1)} \approx 0.2M$), the relative rates of the desolvation reaction follows from Equation (12), 20 and 17:

$$\frac{v_{desol\,(Sol)}}{v_{desol\,(G1)}} = \frac{r^2_{i\,(G1)}}{r^2_{i,\,(Sol)}} \cdot \exp\left(\frac{\Delta G_{desol\,(G1)} - \Delta G_{desol\,(Sol)}}{RT} + \frac{z_e F}{RT}\alpha_{\#1}\Delta\Phi(\alpha_{i,\,(G1,\,0.2\,M)} - \alpha_{i,\,(Sol,\,0.2\,M)})\right) \tag{S19}$$

On basis of DFT calculations, which provide the energies[4] and radii of the (partially) solvated magnesium cations, the reaction rates for the desolvation in the different solvents can be calculated with respect to the rate in G1 [Eq. (S19)] as a function of the potential difference between electrode and electrolyte ($\Delta\Phi_s - \Phi_e$). Thereby, it is important to consider, that always the step with the highest activation energy ($E_{A,desol} \approx \Delta G_{desol}$) will be rate-determining and therefore relevant for the kinetics.

Table S2 Desolvation energy ΔG_{desol} in $kJ\,mol^{-1}$ for the stepwise desolvation of the coordinating oxygen atoms in G1 and G2[4]

CN Mg^{2+}	$6 \rightarrow 5$	$5 \rightarrow 4$	$4 \rightarrow 3$	$3 \rightarrow 2$	$2 \rightarrow 1$	$1 \rightarrow 0$
G1	92	71	100	77	102	84
G2	74	115	82	75	97	82

From the cycling of symmetric magnesium cells (Figure 3) and CV measurements (Figure 4) it is known, that the (partial) desolvation of the magnesium cation is significantly faster in G2 than in G1. From figure S2 it can be seen, that this is only fulfilled for an intermediate with CN=5, with is consistent to the results of the more detailed DFT analysis (Figure 5)

Table S3 Radii r_i in Å for all (partially) desolvated intermediates in G1 and G2

CN Mg^{2+}	6	5	4	3	2	1	0
G1	4.391	5.549	4.511	5.489	4.235	4.490	0.80
G2	4.433	5.855	6.060	4.610	5.761	5.900	0.80

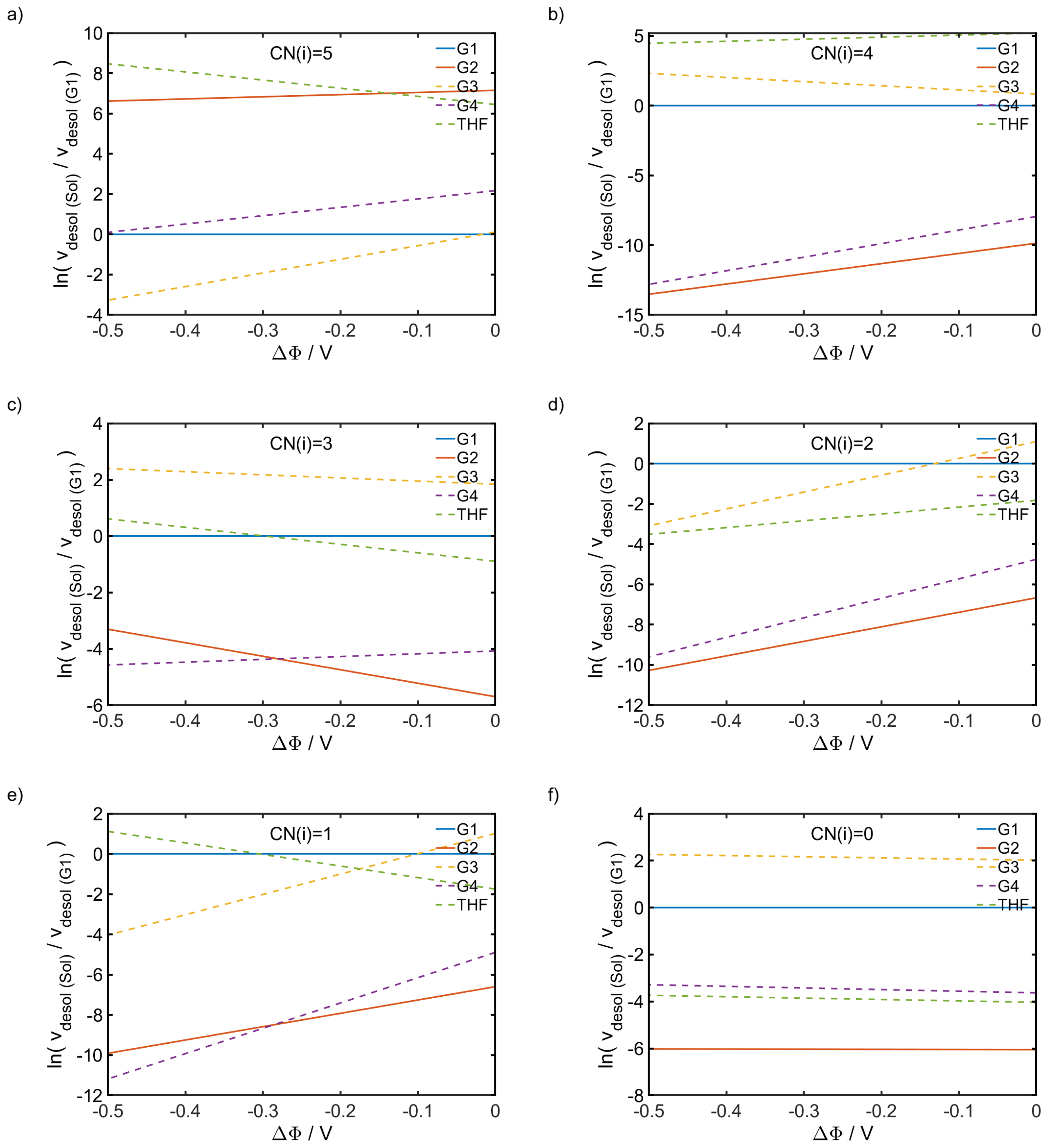

Figure S2 Relative rates of the desolvation for 0.2 M $Mg[B(hfip)_4]_2$ in different solvents for (partially) desolvated intermediates with coordination numbers CN(i) between 5 (a) and 0 (f).

S2.2 Influence of the double layer on the desolvation

In general, the potential decay in the double layer and consequently α_i is mainly determined by the dielectric constant of the solvent and the size of the fully solvated magnesium cation as well as the partially desolvated intermediate [Eqs. (21)-(23)]. Consequently, a solvent with a higher dielectric constant and a sterically more demanding solvation shell of the fully solvated magnesium cation are favorable for the desolvation reaction. However, latter will usually lead to a larger partially desolvated intermediate, which in turn reduces the beneficial effect of the double layer on the desolvation process. Moreover, the electrolyte concentration close to the electrode will also have an important impact on the double layer (Figure S3). Thereby, a lower salt concentration will lead to a higher value for α_i. Therefore, transport limitations in the electrolyte may enhance the desolvation reaction although a lower concentration of the active specie usually slows down the reaction [Eq. (12)]. However, the potential difference between the electrode and electrolyte also has an important impact on the reaction rate, as well as on α_i itself (Figure S2).

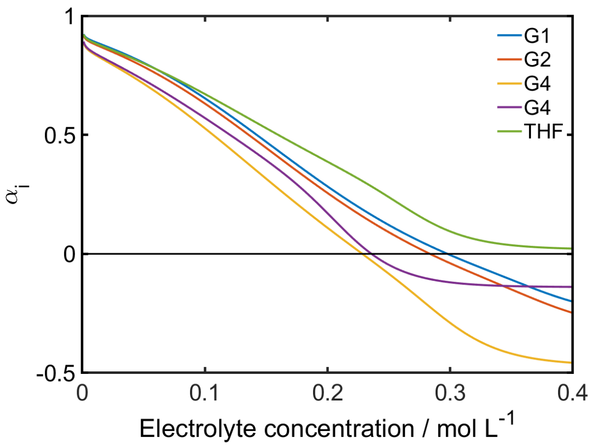

Figure S3 Concentration-dependence of α_i for different solvents.

Figure S3 shows, that our simple model for the electrochemical double layer [Eqs. (21)-(23)] results in a quite strong concentration dependence of α_i. Moreover, it predicts negative values already at intermediate concentrations. Consequently, this approach [Eqs. (21)-(23)] is definitely not able to quantitatively represent the influence of the concentration on the double layer correctly and a more sophisticated model of the double layer needs to be developed. However, the qualitative trend (decrease of α_i with increasing concentration) seems to be reasonable. Therefore, this simple description of the electrochemical double layer might be a sufficient approach especially for battery operation at small current densities, where only small concentration gradients occur. In Table 2 the values of α_i are summarized for the different solvents and an electrolyte concentration of 0.2 M. For G1 the simple double layer model predicts a value for α_i (0.283), which is very similar to the value of the symmetry factor determined for a Butler-Volmer type equation (0.31 [5]). This indicates that our approach to determine α_i leads to reasonable values at least for an 0.2 M electrolyte at low current densities. But more important than the exact values of α_i is the qualitative trend for the different solvents. As assumed in the prior section, the influence of the electrochemical double layer on the desolvation and, therefore, the parameter α_i seems to be comparable for G1 and G2 (Figure S3). This is the result of the quite similar dielectric constant and size of the solvated cations in these two glymes (Table 1). In contrast, the α_i is significantly smaller for the two longer glymes G3 and G4 (Figure S3 and Table 2). Consequently, for these two solvents the favourable effect of

a higher dielectric constant and bulkier solvation shell (Table 1) is destroyed by the large radius of the partially desolvated intermediate (Table 2). In the case of THF the size of the intermediate is very similar to the one in G1 and G2. Therefore, the higher dielectric constant and the significantly larger radius of the fully solvated magnesium cation lead to a larger value for α_i. All in all, a small partially desolvated intermediate (CN=5) seems to be favourable for the desolvation reaction. For the five analyzed solvents the electrochemical double layer can support the desolvation the most in THF and the least in G3. Moreover, this support is significantly stronger in G1 and in G2 than in G4.

S2.3 Rate constants of the desolvation and oxidation reactions

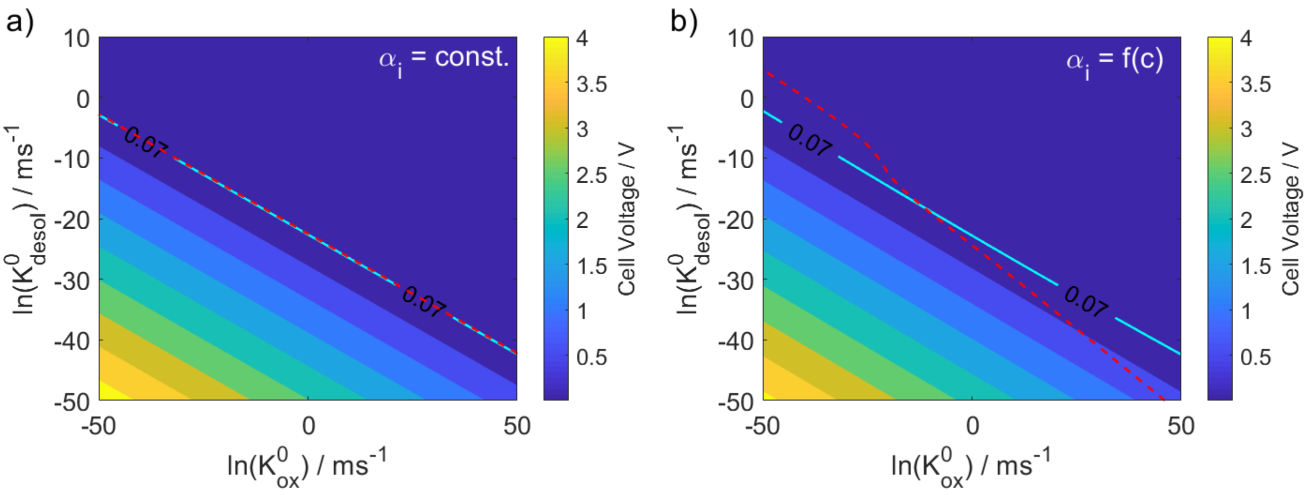

Figure S4 Parameter study: Influence of the rate constants K^0_desol and K^0_ox on the cell voltage for a 0.2 M $Mg[B(hfip)_4]_2$ / G1 electrolyte, a current density of 0.1 mA cm^{-1} and a constant (a) / concentration-dependent (b) value for α_i. The blue line indicates the experimentally observed value of 0.07 V (Table 4), whereas the dashed red line marks the corresponding ratio between the overpotentials of plating and stripping of 1.64 (Figure 7).

S3 Comparison of the simulation results to the experimental data

S3.1 Influence of the solvent

During operation of magnesium batteries a conditioning process is often observed, which leads to a significant decrease of the overpotential during the first cycles.[6–10] This behaviour is caused by the removal of residual passivating magnesium oxide, the increase of the electrode surface area and a possible SEI formation[11,12]. All these surface processes affect the potential of the magnesium working as well as the magnesium reference electrode and are most prominent for freshly prepared electrodes. Although magnesium electrodes were prepared according to the same procedure to remove the native passivation layer and the water content of the different solvents was checked, the surface morphology might vary between the individual magnesium electrodes. Therefore, the absolute overpotentials differ between the first and the reproductive measurement, especially during the first cycles (Figure 8 and S5). Moreover, due to the conditioning process significantly lower and more stable overpotentials are observed for all solvents, when the polarization with the current rate of $0.1\,mA\,cm^{-2}$ is repeated after cycling at higher current densities (Figure S5b). Since the conditioning is not considered in the simulations, the absolute values of the overpotentials including the total cell voltage might not be perfectly comparable to the measured values. Therefore, this work focuses on the general, qualitative trends between the different solvents, which are clearly observable in all measurements (Figure 8 and S5).
Another effect of the conditioning is that the OCV potential shifts significantly during some of the measurements (Figure 8 and S5). This behaviour is differently pronounced for each of the assembled cells, since the surface morphology of the magnesium working and reference electrodes can differ even through similar and careful preparation. Since the OCV potential is regarded to be more reliable after a few cycles, the experimental data is corrected by the OCV after the first ten cycles. Consequently, the initial potentials in Figure 8 and S5 are different from 0 V as well as different between the four solvents.
In order to compare the experimental data with the simulation results the last of the ten cycles, which is least affected by conditioning, is used to determine the ratio between the overpotentials of the two half cell reactions as well as the overall cell voltage. Additionally, the evaluated values are referred to the OCV measured directly before the direction change of the electric current (Figure S9) and the standard deviation of the experimental values is considered throughout the whole evaluation. However, the main parameter to describe the impact of the desolvation on the magnesium deposition, is the ratio between the half cell potentials, which is a relative value. In contrast to the absolute overpotentials, the ratio between the overpotentials during plating and stripping was found to be very similar for the original and the reproductive measurement as well as compared to the values after extensive conditioning (Figure 8 and S5). Therefore, the experiments as well as the simulations allow to study the influence of the desolvation on the magnesium deposition kinetics qualitatively (compared to G1).

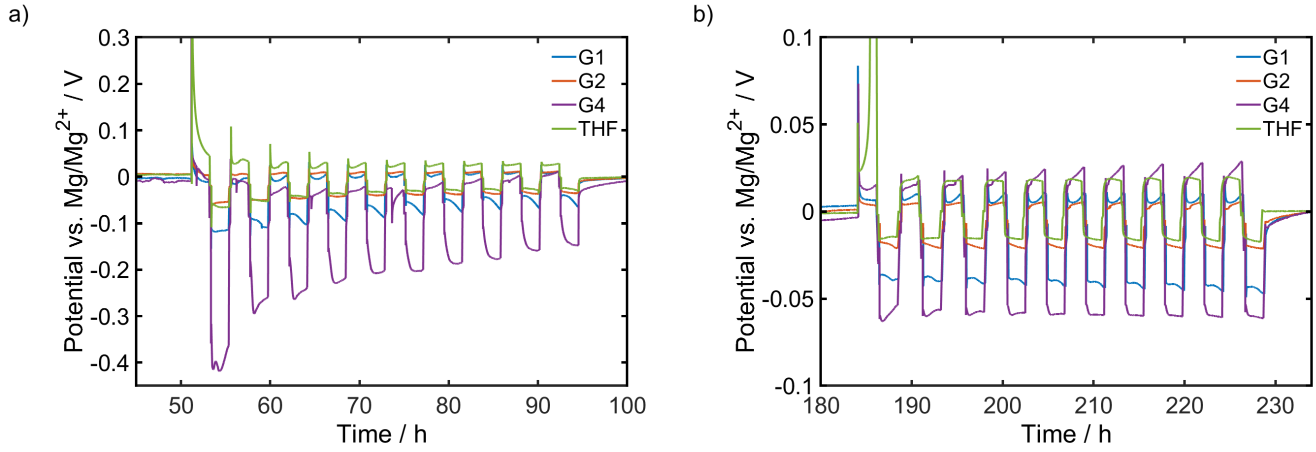

Figure S5 Reproductive measurement: Cycling of symmetric magnesium cells with a magnesium reference electrode and a 0.2 M $Mg[(hfip)_4]_2$ electrolyte based on different solvents at a current density of 0.1 mA cm^{-1} before (a) and after (b) cycling at higher current densities.

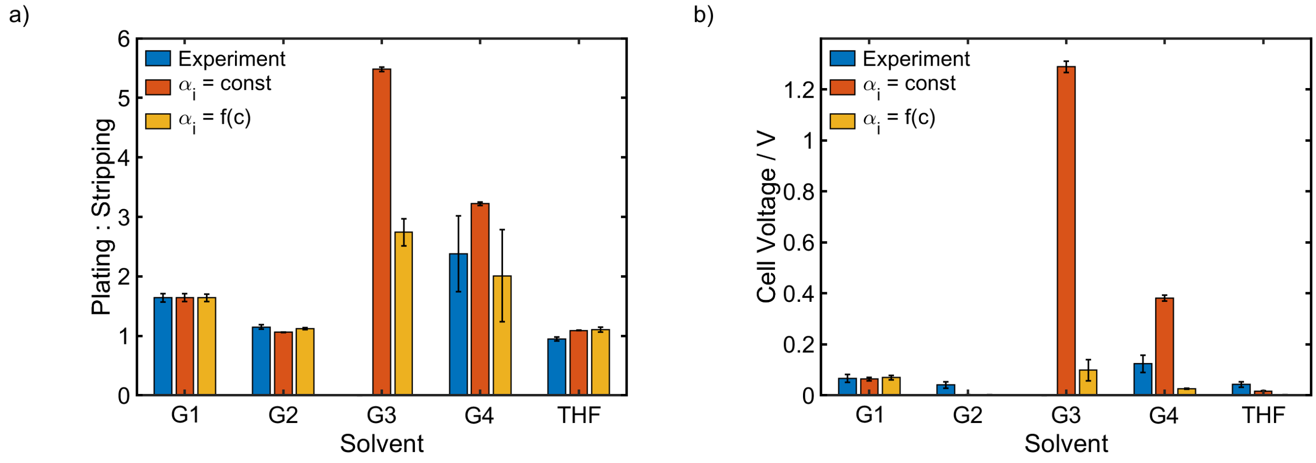

Figure S6 Comparison between experimental data and simulation results for the asymmetry of the overpotentials during plating and stripping (a) and the cell voltage (b) of symmetric magnesium cells with a 0.2 M $Mg[B(hfip)_4]_2$ electrolyte based on different solvents at a current density of 0.1 mA cm^{-1} including the predictions for G3.

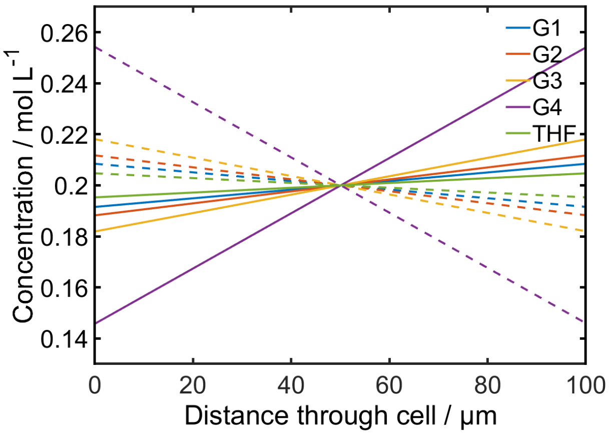

Figure S7 Concentration gradients in symmetric magnesium cells (cathode at 0 µm, anode at 100 µm) with 0.2 M $Mg[(hfip)_4]_2$ electrolyte based on different solvents during charge (dashed) and discharge (solid) at a current density of $0.1\,mA\,cm^{-1}$.

Table S4 Kinetic parameters in G4 for partially desolvated intermediates with different coordination numbers

Parameter	CN=5	CN=4	CN=3
w	0	0	1
r_i [Å]	7.694	7.782	6.865
ΔG_{desol} [kJ mol^{-1}]	85	109	109
$k^0_{desol(Sol)}/k^0_{desol(G1)}$	16.8	$1.1{\cdot}10^{-3}$	$1.1{\cdot}10^{-3}$
$c_{i,max(Sol)}/c_{i,max(G1)}$	0.520	0.508	0.653
$K^0_{desol(Sol)}/K^0_{desol(G1)}$	8.7	$5.3{\cdot}10^{-4}$	$6.9{\cdot}10^{-4}$
$\alpha_{i\,(0.2\,M)}$	0.177	0.167	0.265

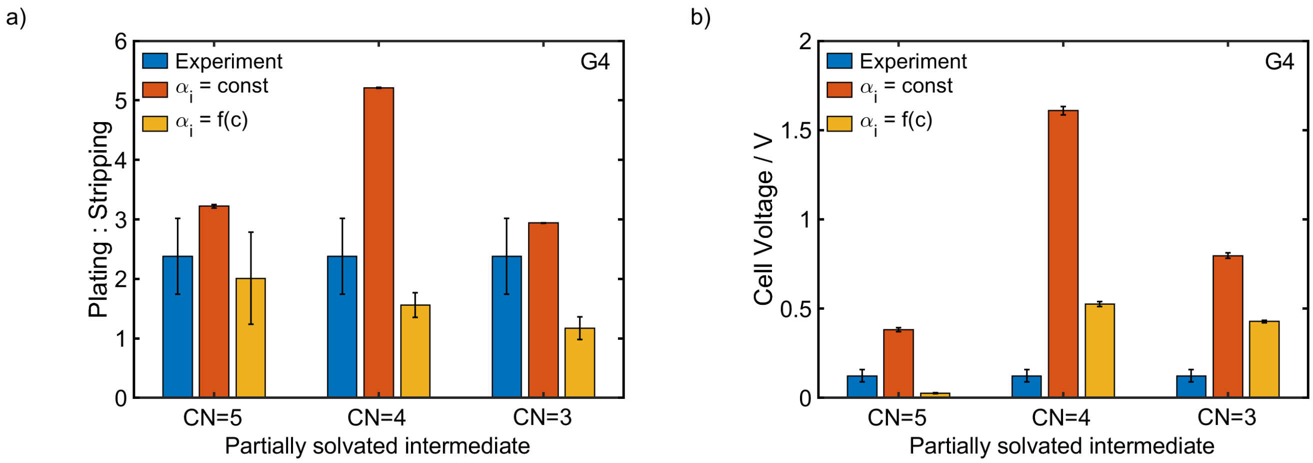

Figure S8 Comparison between experimental data and simulation results for the asymmetry of the overpotentials during plating and stripping (a) and the cell voltage (b) of symmetric magnesium cells with a 0.2 M $Mg[(hfip)_4]_2$ / G4 at a current density of 0.1 mA cm^{-1}. Thereby partially desolvated intermediates with different coordination numbers are analyzed. The according parameters for the kinetic model are summarized in Table S4.

S3.2 Influence of the current density

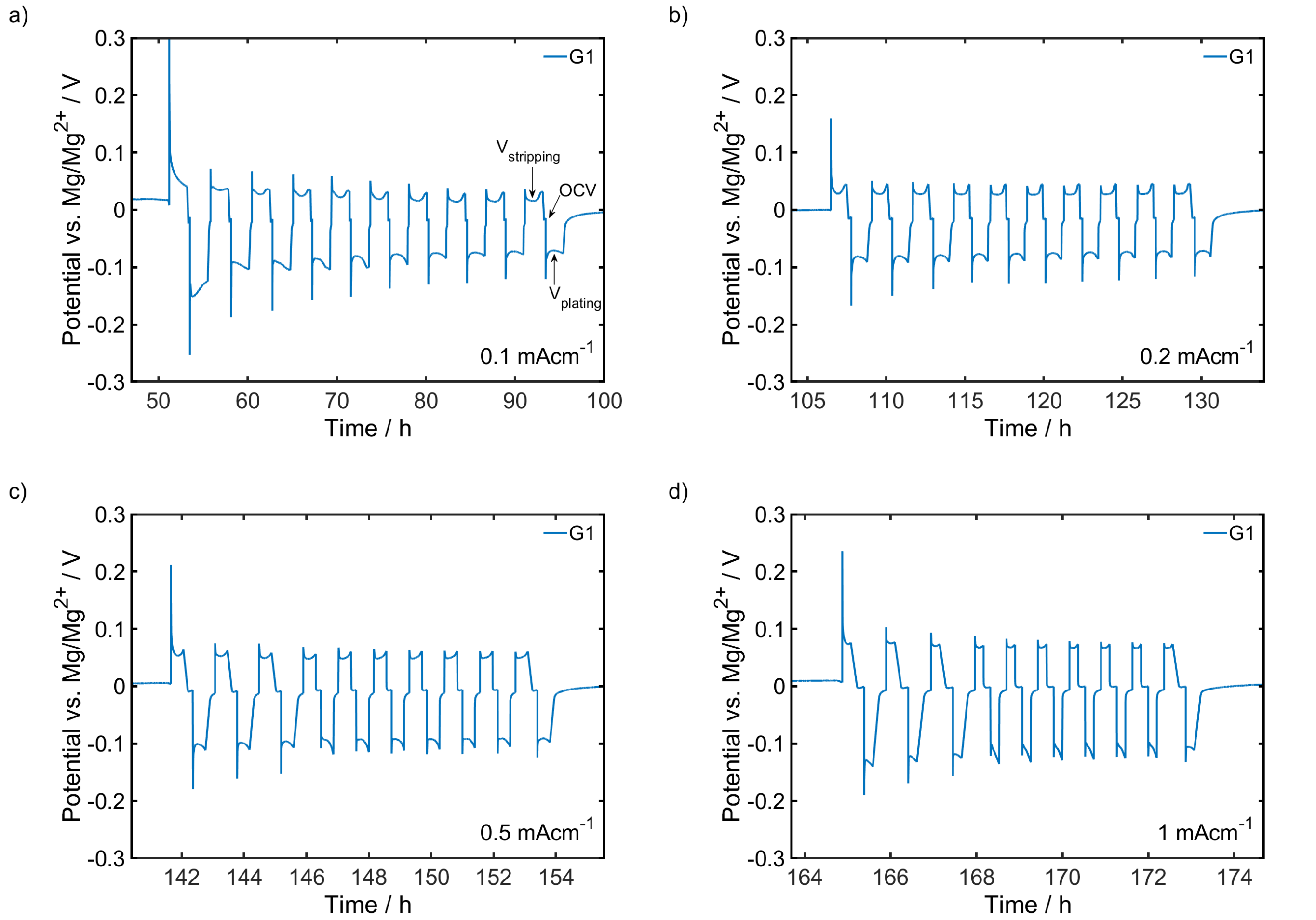

Figure S9 Cycling of symmetric magnesium cells with a magnesium reference electrode and a 0.2 M $Mg[(hfip)_4]_2$ / G1 electrolyte at different current densities. a) additionally shows, how the ratio between the half cell potentials is evaluated.

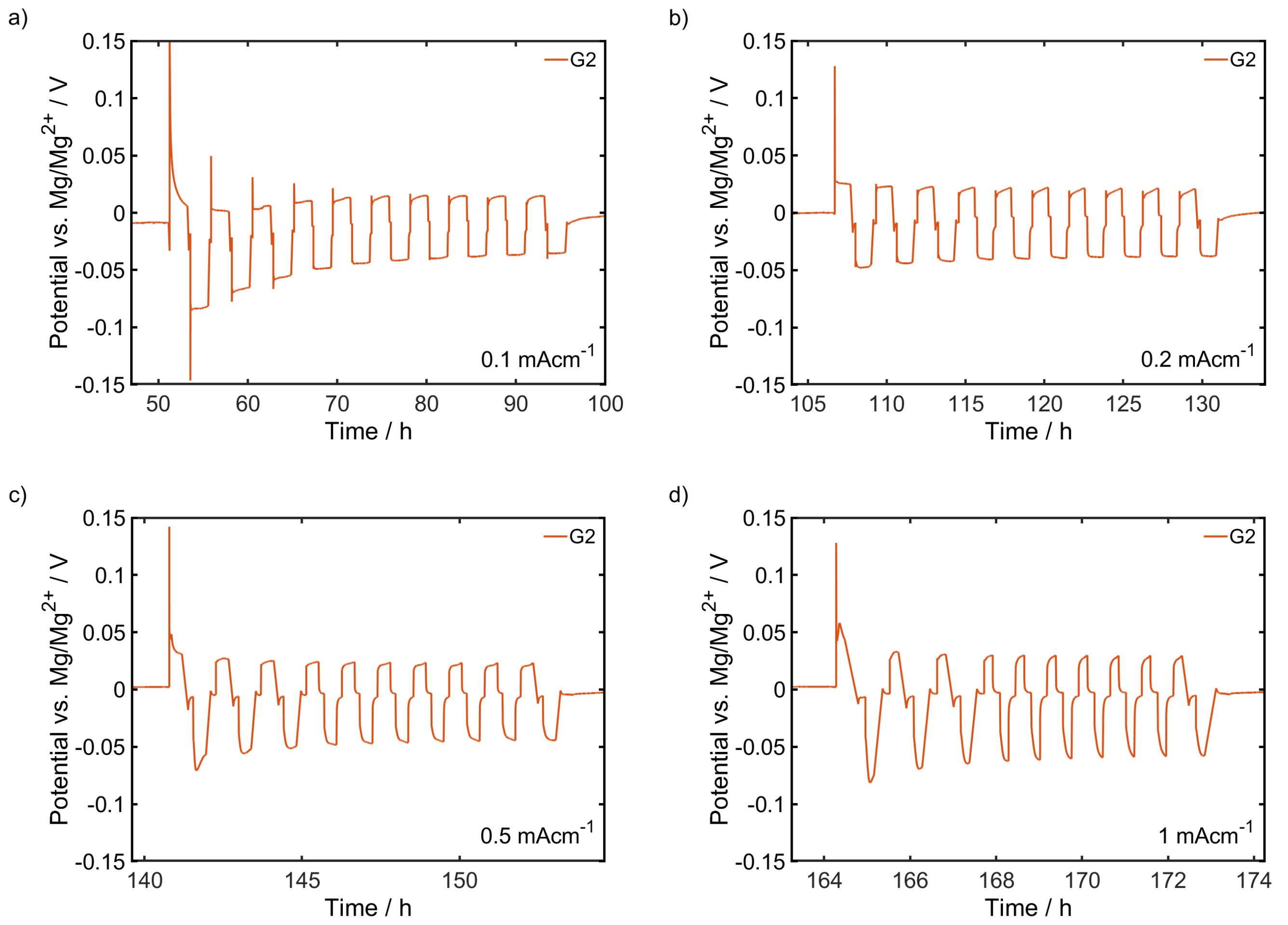

Figure S10 Cycling of symmetric magnesium cells with a magnesium reference electrode and a 0.2 M $Mg[(hfip)_4]_2$ / G2 electrolyte at different current densities.

Table S5 Cell voltage and its asymmetry for 0.2 M $Mg(B[hfip]_4)_2$ / G2 at different current densities.

Current density [mA cm^{-2}]	Plating : Stripping	Cell Voltage [V]
0.1	1.15	0.041
0.2	1.34	0.056
0.5	1.66	0.077
1.0	1.80	0.084

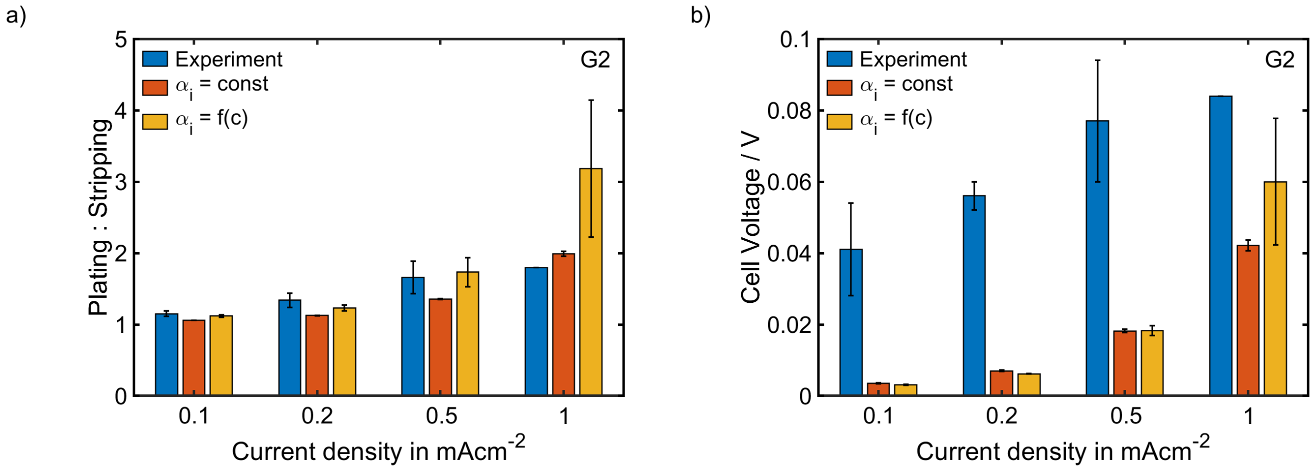

Figure S11 Comparison between experimental data and simulation results for the asymmetry of the overpotentials during plating and stripping (a) and the cell voltage (b) of symmetric magnesium cells with a 0.2 M $Mg[(hfip)_4]_2$ / G2 electrolyte at different current densities.

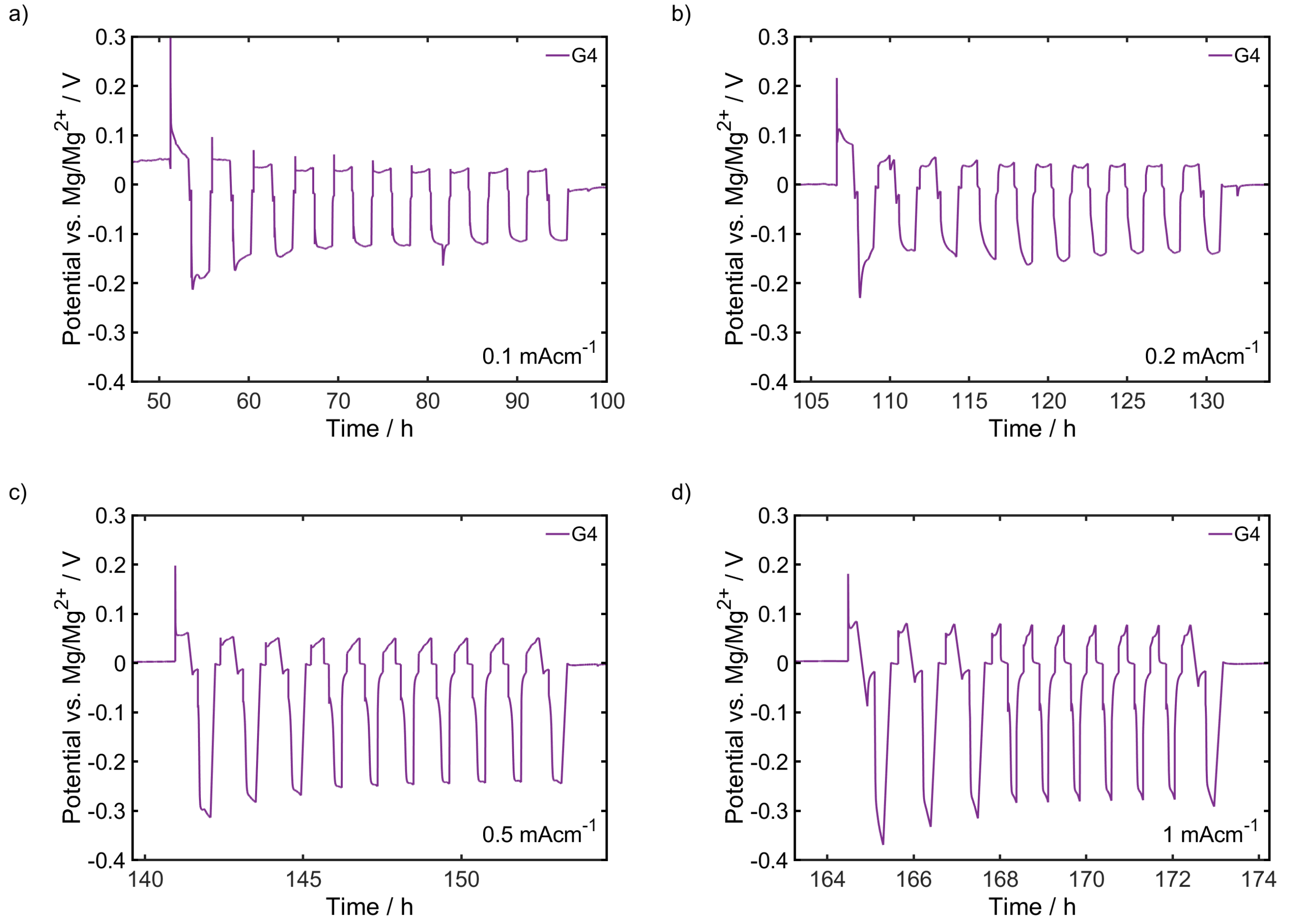

Figure S12 Cycling of symmetric magnesium cells with a magnesium reference electrode and a 0.2 M $Mg[(hfip)_4]_2$ / G4 electrolyte at different current densities.

Table S6 Cell voltage and its asymmetry for 0.2 M $Mg(B[hfip]_4)_2$ / G4 at different current densities.

Current density [mA cm^{-2}]	Plating : Stripping	Cell Voltage [V]
0.1	2.38	0.124
0.2	3.11	0.185
0.5	3.58	0.281
1.0	3.67	0.336

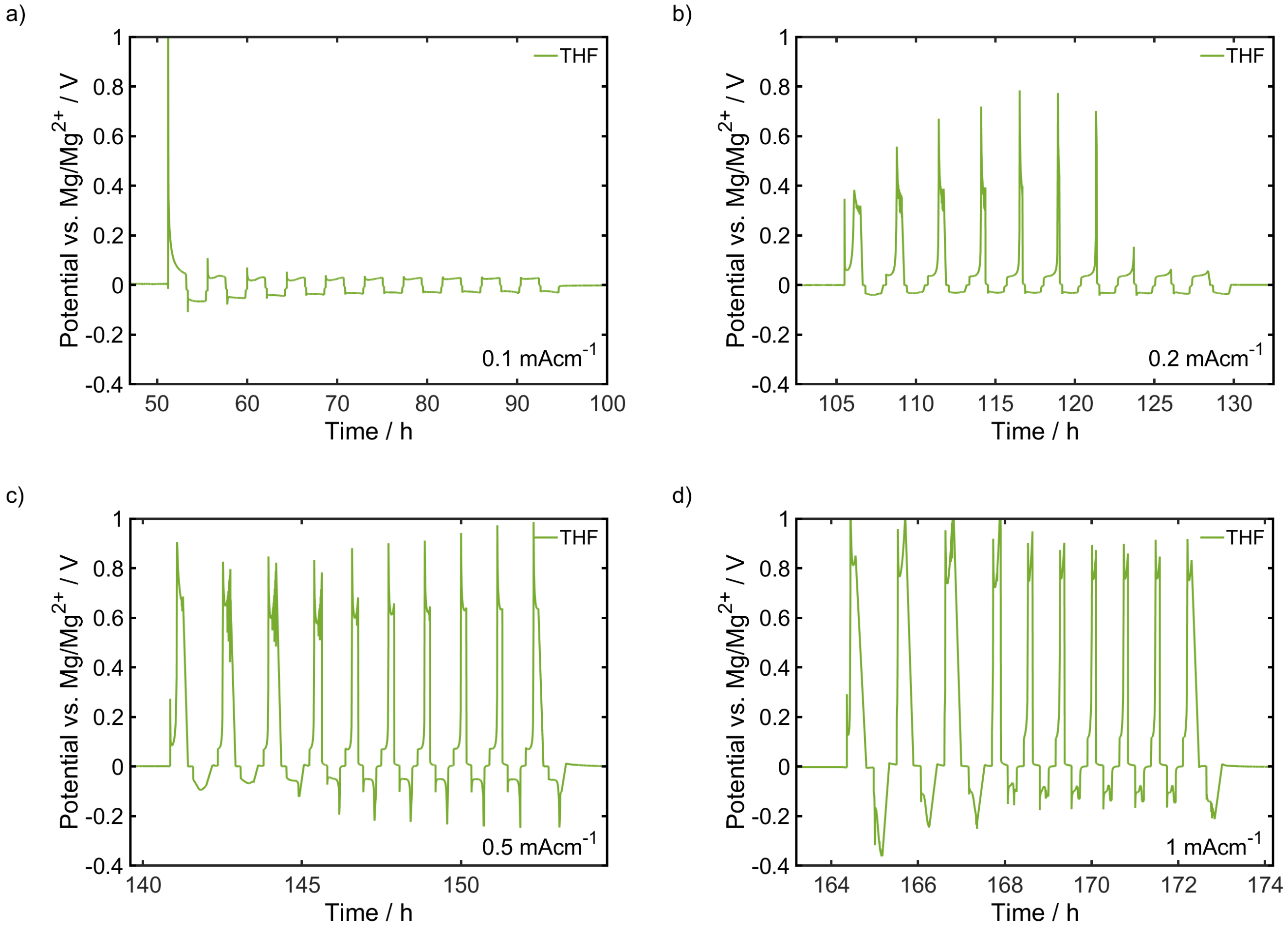

Figure S13 Cycling of symmetric magnesium cells with a magnesium reference electrode and a 0.2 M $Mg[(hfip)_4]_2$ / THF electrolyte at different current densities.

In the case of the THF based electrolyte, the overpotential during stripping is extremely high at current densities >0.1 mA cm^{-2} (Figure S13). Since a higher current density leads to a higher potential difference at the electrode-electrolyte interface, the reason for the surprising increase of the overpotential seems to be the decomposition of THF. Thereby, the critical current density, at which the potential difference can get high enough to cause solvent decomposition, is around 0.2 mA cm^{-2} (Figure S13b). Interestingly, at this current density the extremely high overpotentials only occurs during the first cycles. During the last cycles the overpotential for magnesium stripping is reasonable. This behavior can be explained by the increase of the electrode surface area during cycling, which generally leads to an exponential decay of the overpotentials with the number of cycles (Figure 8). Thereby, this decay is most pronounced in the first few cycles. Afterwards the overpotential only decays very slightly and approaches an almost stable value with increasing cycle number. However, there is still a small increase of the electrode surface during the cycling at 0.2 mA cm^{-2}, which might cause a drop of overpotential, which is required for magnesium dissolution, under the critical value of the potential difference, at which solvent decomposition takes place.

In general, two decomposition products of THF are known in magnesium electrolytes: γ-butyrolactone[13] and poly-THF[9,14]. Both can be generated by oxidation of THF. An oxidative solvent decomposition can be expected to take place at the anode. Since this is also the electrode, at which the magnesium is stripped, this would explain, why the overpotential is only increased

during magnesium dissolution and not during magnesium deposition (Figure S13). However, the solvent decomposition does not seem to form a passivation film on the electrode surface. This can be seen by the fact, that after the direction of the current is changed, magnesium can be deposited without an significantly increased overpotential. Moreover, the overpotentials of both half cell reactions come back to normal values, when the potential difference at the electrode does not enable the THF decomposition anymore (Figure S13b). For poly-THF it is know, that it does not passivate the electrode, while it can cause an increase of the overpotential.[9,14] Furthermore, the partially negatively charged oxygen atoms of the THF decomposition products might form an adsorption layer on the positively charged anode, which hinders the magnesium dissolution. As soon as the polarization of the cell is changed, the adsorption layer might not be stable on the now negatively charged electrode, so that the magnesium deposition is not significantly hindered by the decomposition products. All in all, THF seems not to be a suitable solvent for the chloride-free $Mg[B(hfip)_4]_2$ electrolyte. However, the presence of electron-donor anions (e.g. chlorides) in the Mg^{2+} solvation shell might inhibit the THF decomposition.[15,16]

S3.3 Butler-Volmer kinetics

The Butler-Volmer equation is a very common description for the reaction kinetics at the electrode-electrolyte interface.

$$i_{se} = z_+F \cdot k_{BV} \cdot c_e^{(1-\alpha_{BV})} c_s^{\alpha_{BV}} \cdot \left(\exp\left[\frac{z_+F}{RT}(1-\alpha_{BV})\eta_s\right] - \exp\left[-\frac{z_+F}{RT}\alpha_{BV}\eta_s\right]\right) \tag{S20}$$

whereby k_{BV} is the corresponding rate constant and α_{BV} is the symmetry factor, which enables to consider that plating and stripping can contribute differently to the overall overpotential η_s. Latter is defined as deviation of the experimentally observed potential from the corresponding standard half-cell reduction potential $\Delta\Phi_0^{eq}$ given by Nernst's equation [Eq. (S16)].

$$\eta_s = \Phi_s - \Phi_e - \Delta\Phi^{eq} \tag{S21}$$

A parameter study can give insights on the values for k_{BV} and α_{BV}, which fit to the experimental observations in the analyzed ethereal solvents (Figure S14). The results are summarized in Table S7. For G1 the determined characteristic parameters of the Butler-Volmer equation fit well to previously reported values.[5] For other solvents corresponding data is not available in the literature. However, the comparison of k_{BV} and especially α_{BV} in different solvents is interesting. The symmetry factor indicates to what extend desolvation of the magnesium cation hinders the deposition reaction. Thereby, a lower value of α_{BV} can be interpreted as a more severe influence of the desolvation on the battery performance. As expected, a higher ratio of the plating and stripping half cell potentials is generally connected to a lower value of α_{BV}, whereas a lower rate constant k_{BV} of the electrochemical reaction leads to a higher cell voltage (Figure S14). Note, that the different transport properties in the solvents also influence the correlation between the Butler-Volmer parameters and the plating : stripping ratio as well as the cell voltage (Figure S14).

Table S7 Parameters of the Butler-Volmer equation for $Mg[(hfip)_4]_2$ in different solvents determined by parameter study (Figure S14).

solvent	k_{BV} [m s^{-1}]		α_{BV}	
	mean	sdv	mean	sdv
G1	$5.1 \cdot 10^{-9}$	$1.7 \cdot 10^{-9}$	0.359	0.017
G2	$7.9 \cdot 10^{-9}$	$2.9 \cdot 10^{-9}$	0.464	0.014
G4	$2.5 \cdot 10^{-9}$	$1.3 \cdot 10^{-9}$	0.331	0.053
THF	$6.3 \cdot 10^{-9}$	$1.7 \cdot 10^{-9}$	0.533	0.010

Unfortunately, the symmetry factor of the Butler-Volmer equation [Eq. (S20)] is a purely phenomenological parameter and no correlation between the desolvation energies ΔG_{desol} (Table 2) and the values of α_{BV} (Table S7) can be observed. Consequently, it is not possible to predict the impact of the desolvation on the battery performance on the basis of calculations with Butler-Volmer kinetics.

Moreover, ion pairing and clustering is commonly observed in magnesium electrolytes especially in chloride containing ones. However, it is not straightforward to consider multiple electrochemically active species with the Bulter-Volmer approach, which is based on the Nernst relation [Eq. (S16)]. It can't simply be assumed, that the equilibrium potential will be determined by the total magnesium concentration since ion pairs or clusters can show a completely different reduction behaviour

than solvated magnesium cations. In contrast it is very simple to extend our herein presented kinetic model to electrolytes with multiple magnesium species [Eq. (1)]. Thereby, the parametrization of the model can be done purely on the basis of DFT calculations. Note, that as described in section S1.2.3, it might be necessary to consider an effective plating rate constant instead of a desolvation constant [Eqs. (S17) and (S18)] for active species, which are desolvated quite easily.

Table S7 clearly shows, that in contrast to many other systems the anodic and cathodic reactions can be very asymmetric in magnesium electrolytes. Therefore, it is not possible to capture the experimentally observed ratios between the half cell potentials with the common assumption of $\alpha_{BV} \approx 0.5$ (Figure S15a and S15c). With the determined Butler-Volmer parameters (Figure S14 and Table S7) the behavior of the plating : stripping ratio can be reproduced quite well for G2 (Figure S15c). However, in this solvent the symmetry factor is close to the ideal value of 0.5 and therefore the desolvation has only a minor impact on the magnesium deposition in G2. This is different in the case of G1: Even though the predicted values for the ratio of the half cell potentials at higher current densities are quite close to the experimental ones for an α_{BV} of 0.36, the qualitative trend, namely the decrease of the plating : stripping ratio with increasing current densities, can not be captured with simulations based on Butler-Volmer kinetics (Figure S15a).

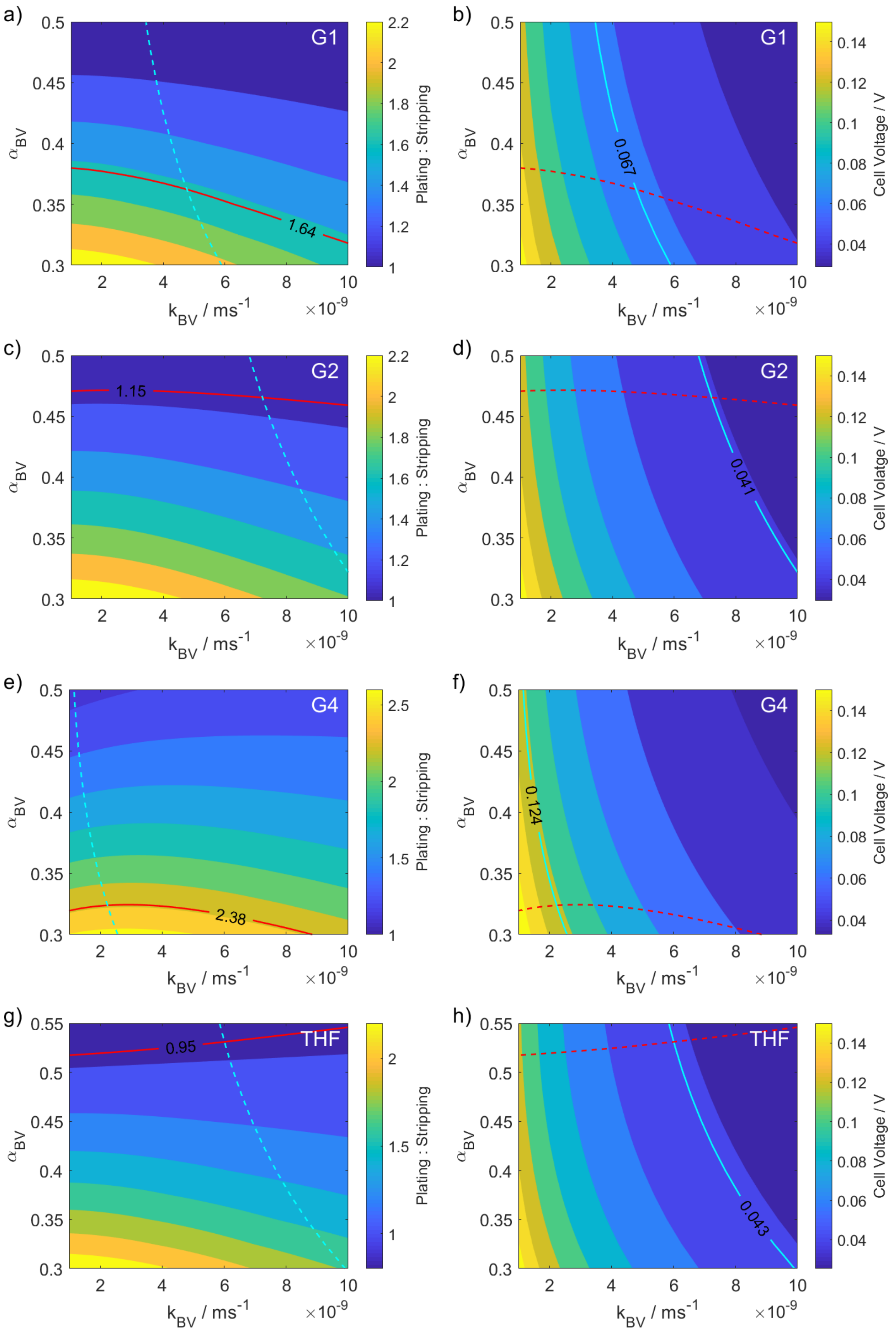

Figure S14 Parameter study with Butler-Volmer kinetics: Influence of the rate constant k_{BV} and the symmetry factor α_{BV} on the ratio between the overpotentials of plating and stripping (left) and the cell voltage (right) for a 0.2 M $Mg[(hfip)_4]_2$ in different solvents and a current density of 0.1 mA cm^{-1}. The red line indicates the experimentally observed value of the plating : stripping ratio, whereas the blue line marks the corresponding measured cell voltage (Table 4).

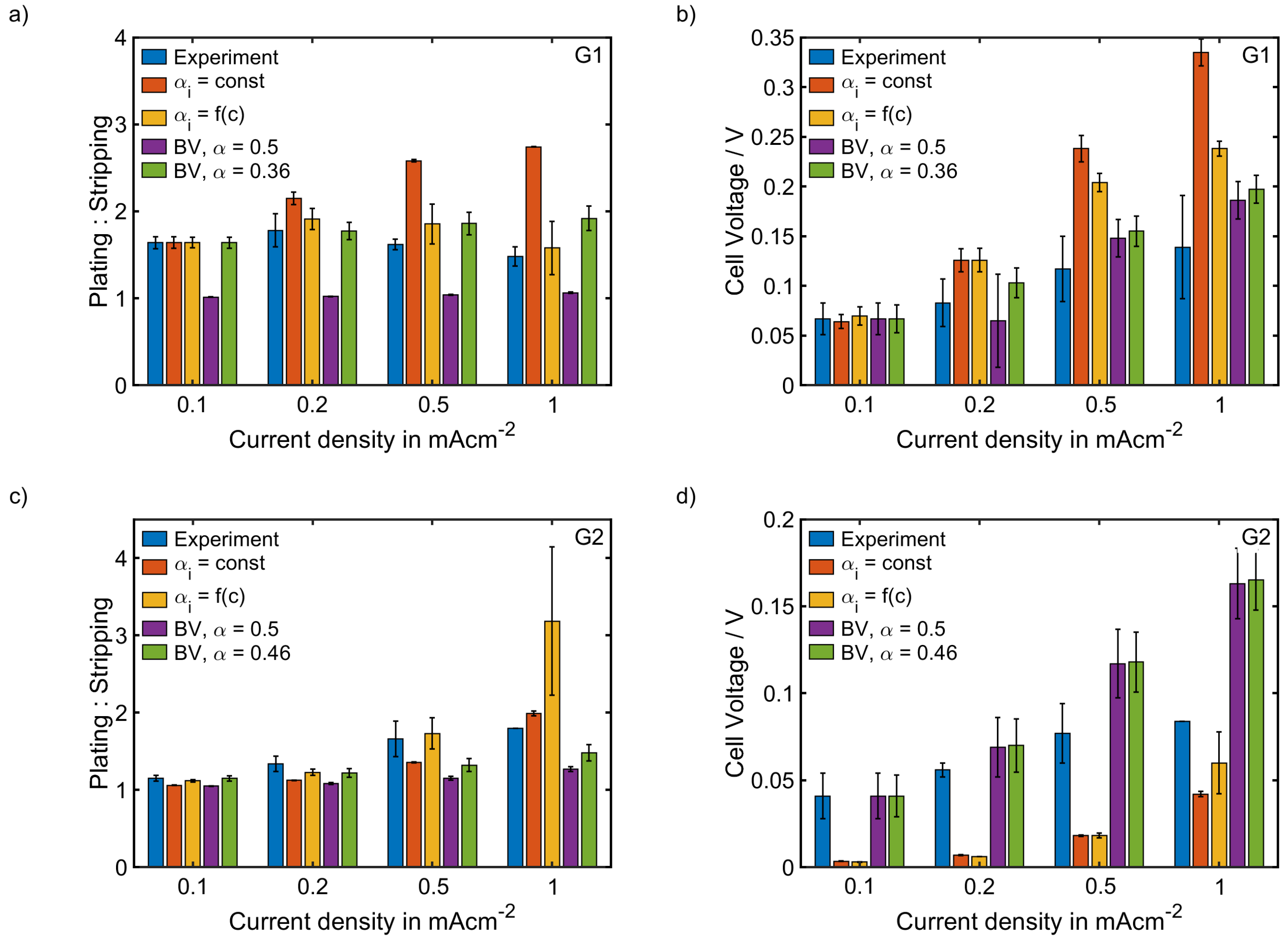

Figure S15 Comparison between experimental data and simulation results from different kinetic models for the asymmetry of the overpotentials during plating and stripping (left) and the cell voltage (right) of symmetric magnesium cells with a 0.2 M $Mg[(hfip)_4]_2$ in G1 (top) and G2 (bottom) at different current densities.

S3.4 Influence of the desolvation energy

To get more insight on the impact of the initial desolvation on the magnesium deposition a parameter study is done, in which the corresponding desolvation energy $\Delta G_{\mathrm{desol}}$ and therefore the rate constant K^0_{desol} is varied for the different solvents. The results are shown in Firgure S16. It can be seen, that the ratio between the plating and stripping overpotentials changes sigmoidal with an increasing desolvation energy, so that two limiting cases for low and high $\Delta G_{\mathrm{desol}}$ can be identified. For desolvation energies above 110 $\mathrm{kJmol^{-1}}$ the plating : stripping ratio converges towards an upper limit, which is determined by the double layer parameter α_i (Figure S17b). Thereby, the limiting value at high $\Delta G_{\mathrm{desol}}$ increases exponentially with a decreasing value of α_{i}. Surprisingly, the lower limit of the plating : stripping ratio can vary from 1. Consequently, the ratio of the half cell potentials does not only represent the influence of the desolvation on the plating reaction but also further impacts of the solvent. However, in the cases of G1, G3 and G4 the experimental as well as the predicted plating : stripping ratios are significantly higher than the corresponding lower limit (Figure S6a and S16a). Thus, in these cases the main contribution to the increased plating overpotential can clearly be assigned to the initial desolvation step including the effect of the electrochemical double layer, which can facilitate the desolvation to a certain extent. Interestingly, the lower limit of the plating : stripping ratio correlates with the transport properties of the solvent, more precisely with the diffusion coefficient (Figure S17a) and therefore the solvent viscosity and the size of the fully solvated magnesium cation [Eq. (16)]. Thereby, again an exponential dependence can be observed (Figure S17a). Consequently, a pronounced asymmetry between the half cell potentials can also be observed in solvents, in which the diffusion is extremely slow.

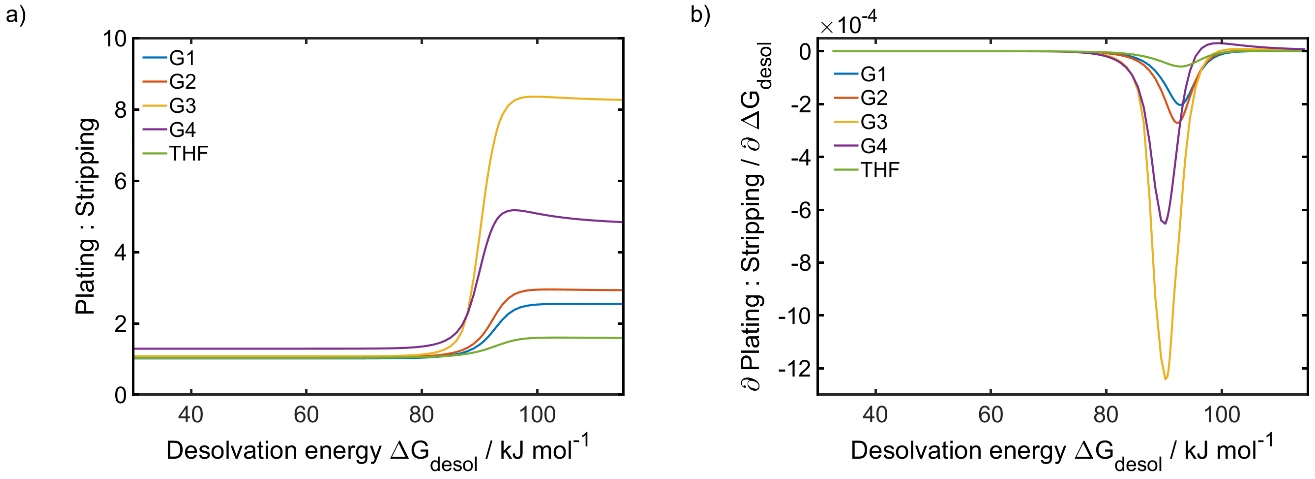

Figure S16 Parameter study for α_{i} =const.: Influence of the desolvation energy on the asymmetry of the overpotentials during plating and stripping for cycling at 0.1 $\mathrm{mA\,cm^{-2}}$ with 0.2 M $\mathrm{Mg[(hfip)_4]_2}$ in different solvents.

A similar impact of the transport on the plating : stripping ratio could also be observed with Butler-Volmer kinetics (Figure S15). For simulations with $\alpha_{\mathrm{BV}} = 0.5$, in which the plating and stripping reaction are assumed to be perfectly symmetric, the plating : stripping ratio is not exactly 1. For G1 the simulation results show a value of 1.01, whereas for G2, in which the diffusion is slightly slower, the plating : stripping ratio is 1.05. Consequently, the increasing impact of the transport at higher current densities, which leads to higher concentration gradients, is mainly responsible for the increase of the plating : stripping ratio with increasing current densities for simulations with

Butler-Volmer kinetics as well as for simulations with $\alpha_i = const.$ (Figure S15).

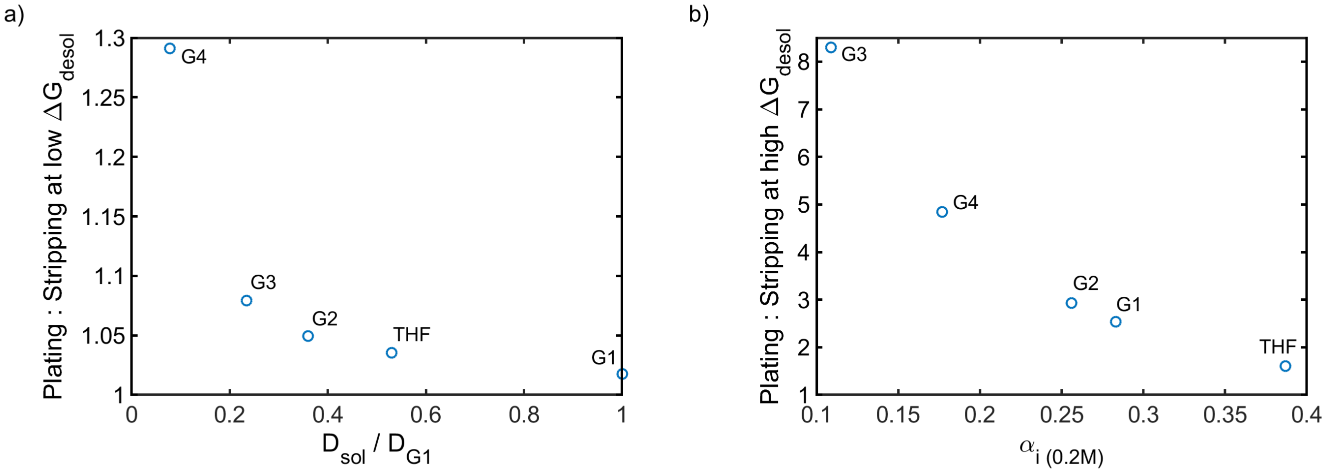

Figure S17 Correlations between the ratio of the half cell potentials at low and high desolvation energies (Figure S16) and the diffusion coefficient (a) as well as α_i (b).

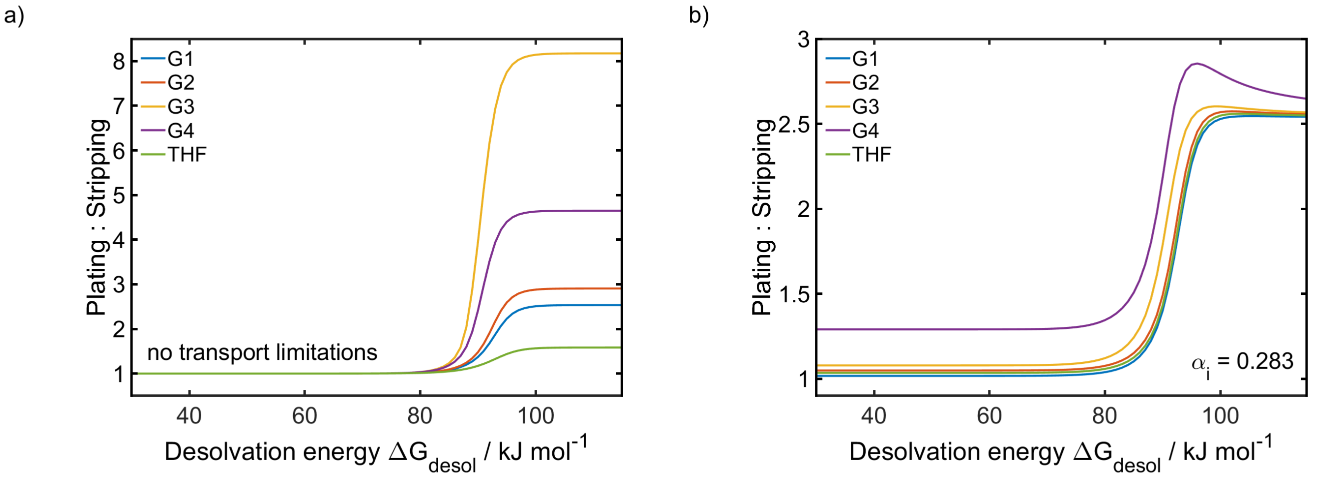

Figure S18 Parameter study for α_i =const.: Influence of the desolvation energy on the asymmetry of the overpotentials during plating and stripping without transport limitations (a) and for a solvent independent α_i (b).

To validate the observed correlations of the plating : stripping ratios at low and high ΔG_{devol} with the double layer parameter α_i and the diffusion coefficient (Figure S17) parameter studies with respect to the desolvation energy are repeated for a solvent independent α_i of 0.283 and for very high transport parameters (Figure S18). Without any transport limitations the overpotentials of plating and stripping become perfectly symmetric for low desolvation energies - independent of the solvent (Figure S18a). Moreover, it can clearly be seen, that the upper limit is defined by the value of α_i (Figure S18b). Thereby, a slow transport causes the convergence of the plating : stripping ratio to take longer. All in all, the additional parameter studies (Figure S18) perfectly confirm the obeserved correlations of the limiting values to transport and double layer properties (Figure S16 andS17). For very low ΔG_{desol} only the transport in the solvent, namely the diffusion, is responsible for the asymmetry of the overpotentials, whereas the impact of the electrochemical double layer on the desolvation become dominating at high desolvation energies.

Independent of further solvent properties, the most significant change of the plating : stripping ratio is observed for a desolvation energy of 90-93 $\mathrm{kJmol^{-1}}$ (Figure S16b). However, the impact of the desolvation on the magnesium deposition can already be observed for lower desolvation energies. Generally, we observe that the adverse effect of the desolvation is almost neglectable when the energy barrier of the initial desolvation is lower than 80 $\mathrm{kJmol^{-1}}$ (Figure S16a). This observation is consistent with the finding, that the magnesium deposition in G2 and THF is not significantly hindered by the desolvation, which only requires 74 and 76 $\mathrm{kJmol^{-1}}$ respectively (Table 2 and Figure 8). Moreover, this also explains the often observed fact, that the presence of chlorides is beneficial for magnesium plating.[7,17,18] For $MgCl^+$ and $MgCl_2$ it was found, that the initial desolvation barrier is lower than 60 $\mathrm{kJmol^{-1}}$ in THF as well as in glymes (G1-G4).[4] Therefore, the desolvation seems not to hinder magnesium deposition in chloride-containing electrolytes. However, intercalation electrodes usually require a full desolvation of the magnesium cation, which is connected to energy barriers above 80 $\mathrm{kJmol^{-1}}$ independent of the solvent and the presence of chlorides.[4] Consequently the desolvation as well as the influence of the electrochemical double layer can not be neglected in the case of magnesium intercalation.

S4 List of symbols

D	Diffusion coefficient
E_A	Activation energy
F	Faraday constant
I	Ionic strength
K^0_ox, K^0_desol	Apparent rate constant of the oxidation (stripping) / desolvation (plating)
M	Molar mass
N_A	Avogadro constant
$\vec{N}_\mathrm{e}$	Particle flux in the bulk electrolyte
R	Universal gas constant
T	Temperature
a	Activity
c_j	Concentration of the specie j
c_max	Maximal concentration of the intermediate in a monolayer on the electrode surface
e	Elementary charge
f_thermo	Thermodynamic factor
i_se	Current density at the electrode-electrolyte interface
$\vec{j}_\mathrm{e,s}$	Electric current in the bulk electrolyte / bulk solid phase
k	rate constant
k^0	Chemical rate constant
k_B	Boltzmann constant
r_e	Hydrodynamic radius of the fully solvated magnesium cation
r_i	Radius of the partially desolvated intermediate
r_ref	Reference length = distance from electrode surface, at which the electric potential of the electrolyte dropped to its bulk value
t_+	Transference number

v_{ox}, v_{desol}	reaction rate of oxidation (stripping) / desolvation (plating)
w	Number of solvent molecules, which are desolvated prior to electron transfer
x	Solvation number of the fully solvated Mg^{2+}
y	Number of released solvent molecules (per cluster)
z_+	Number of transferred electrons
z_j	Charge number of the specie j

α_{BV}	Symmetry factor in the Butler-Volmer equation
α_i	Double layer parameter
$\alpha_{\#1}$, $\alpha_{\#2}$	Symmetry factor of the desolvation / oxidation reaction
γ	Activity coefficient
ΔG_{desol}	Desolvation energy
$\Delta\Phi^{eq}$	Half-cell reduction potential
ε_r	Relative permittivity of the solvent
ε_0	Vacuum permittivity
η	Viscosity
η_s	Overpotential
κ	Ionic conductivity
λ_D	Debye length
μ	Effective chemical potential
μ_j	Chemical potential of the specie j
ρ	Mass density
σ	Electronic conductivity
Φ_e	Electrical potential of the electrolyte
Φ_i	Electric potential experienced by the intermediate
Φ_s	Electrical potential of the electrode

Subscripts s, e, i indicate a quantity of the solid metal electrode, the electrolyte and the partially desolvated intermediate respectively.

References

[1] E. Samson, G. Lemaire, J. Marchand and J. J. Beaudoin, *Comput. Mater. Sci.*, 1999, **15**, 285–294.

[2] A. Latz and J. Zausch, *J. Power Sources*, 2011, **196**, 3296–3302.

[3] J. Drews, T. Danner, P. Jankowski, T. Vegge, J. M. García Lastra, R. Liu, Z. Zhao-Karger, M. Fichtner and A. Latz, *ChemSusChem*, 2020, **13**, 3599–3604.

[4] P. Jankowski, J. M. García Lastra and T. Vegge, *Batteries Supercaps*, 2020, **3**, 1350–1359.

[5] A. F. Chadwick, G. Vardar, S. DeWitt, A. E. S. Sleightholme, C. W. Monroe, D. J. Siegel and K. Thornton, *J. Electrochem. Soc.*, 2016, **163**, A1813–A1821.

[6] Z. Zhao-Karger, R. Liu, W. Dai, Z. Li, T. Diemant, B. P. Vinayan, C. Bonatto Minella, X. Yu, A. Manthiram, R. J. Behm, M. Ruben and M. Fichtner, *ACS Energy Lett.*, 2018, **3**, 2005–2013.

[7] R. Attias, M. Salama, B. Hirsch, Y. Goffer and D. Aurbach, *Joule*, 2019, **3**, 27–52.

[8] S. J. Kang, H. Kim, S. Hwang, M. Jo, M. Jang, C. Park, S. T. Hong and H. Lee, *ACS Appl. Mater. Interfaces*, 2019, **11**, 517–524.

[9] C. J. Barile, E. C. Barile, K. R. Zavadil, R. G. Nuzzo and A. A. Gewirth, *J. Phys. Chem. C*, 2014, **118**, 27623–27630.

[10] M. Winter, *ACS Appl. Mater. Interfaces*, 2019, **11**, 24057–24066.

[11] J. Häcker, C. Danner, B. Sievert, I. Biswas, Z. Zhao-Karger, N. Wagner and K. A. Friedrich, *Electrochim. Acta*, 2020, **338**, 135787.

[12] J. Z. Hu, N. R. Jaegers, Y. Chen, K. S. Han, H. Wang, V. Murugesan and K. T. Mueller, *ACS Appl. Mater. Interfaces*, 2019, **11**, 38689–38696.

[13] C. J. Barile, R. Spatney, K. R. Zavadil and A. A. Gewirth, *J. Phys. Chem. C*, 2014, **118**, 10694–10699.

[14] S. A. Campbell, C. Bowes and R. S. McMillan, *J. Electroanal. Chem.*, 1990, **284**, 195–204.

[15] A. Kopač Lautar, J. Bitenc, T. Rejec, R. Dominko, J. S. Filhol and M. L. Doublet, *J. Am. Chem. Soc.*, 2020, **142**, 5146–5153.

[16] H. D. Yoo, I. Shterenberg, Y. Gofer, G. Gershinsky, N. Pour and D. Aurbach, *Energy Environ. Sci.*, 2013, **6**, 2265–2279.

[17] A. Ponrouch, J. Bitenc, R. Dominko, N. Lindahl, P. Johansson and M. R. Palacin, *Energy Stor. Mater.*, 2019, **20**, 253–262.

[18] P. Canepa, G. S. Gautam, R. Malik, S. Jayaraman, Z. Rong, K. R. Zavadil, K. Persson and G. Ceder, *Chemistry of Materials*, 2015, **27**, 3317–3325.
